# Comparative efficacy and safety of surgical interventions for communicating hydrocephalus: a systematic review and network meta-analysis of randomized controlled trials

**DOI:** 10.3389/fneur.2026.1763131

**Published:** 2026-02-25

**Authors:** Zhihao Zhao, Yang Liu, Weiwei Jiang, Shuangyu Wang, Huijie Yu, Xin Qu, Shangzhi Xiong, Xiaoying Chen, Craig S. Anderson, Tao Liu, Rongcai Jiang

**Affiliations:** 1 Department of Neurosurgery, Tianjin Neurological Institute, State Key Laboratory of Experimental Hematology, Laboratory of Post-Neuroinjury Neurorepair and Regeneration in Central Nervous System Tianjin & Ministry of Education, Tianjin Medical University General Hospital, Tianjin, China; 2 Department of Rehabilitation Medicine, Zhujiang Hospital, Southern Medical University, Guangzhou, China; 3 Department of Cardiology, Tianjin Medical University General Hospital, Tianjin, China; 4 Department of Neurosurgery, Xuanwu Hospital, Capital Medical University, Beijing, China; 5 Faculty of Medicine, The George Institute for Global Health, University of New South Wales, Sydney, NSW, Australia

**Keywords:** choroid plexus cauterization, communicating hydrocephalus, endoscopic third ventriculostomy, lumboperitoneal shunt, network meta-analysis, ventriculoperitoneal shunt

## Abstract

**Objectives:**

Surgical intervention is the standard treatment for communicating hydrocephalus (CH), a condition involving cerebrospinal fluid (CSF) accumulation in the ventricles without a blockage. The optimal surgical approach for CH remains uncertain, with clinical decisions varying by patient characteristics and institutional practices. This study aims to compare the efficacy and safety of surgical interventions for CH.

**Methods:**

In this systematic review and network meta-analysis (NMA), we searched PubMed, Embase, the Cochrane Central Register of Controlled Trials, Web of Science, ClinicalTrials.gov, China National Knowledge Infrastructure (CNKI), Wanfang, Vip, China Biomedical Literature, and the Chinese Clinical Trial Registry (ChiCTR) from inception to September 24, 2024, for randomized controlled trials (RCTs). Primary outcomes were efficacy (favorable outcome) and safety (complications). Secondary outcomes included revision, infection, seizures, operation time (minutes), and duration of hospitalization (days). Bayesian NMAs synthesized the data, and the certainty of evidence was assessed using the confidence in NMA (CINeMA) framework. Surface under the Cumulative Ranking Curve (SUCRA) values were generated to rank the treatments. This study is registered with PROSPERO (CRD42024585931).

**Results:**

Of 4,159 citations identified by our search, 34 trials (2,528 participants) met the inclusion criteria. For efficacy, lumboperitoneal shunt (LPS) [risk ratio (RR) 1.18, 95% credible interval (CrI) 1.13–1.23; high certainty] and LPS with laparoscope (LPS + LS) (RR 1.27, 95% CrI 1.18–1.39; high certainty) were more effective than ventriculoperitoneal shunt (VPS). Both LPS and LPS + LS outperformed endoscopic third ventriculostomy (ETV) and ETV with choroid plexus cauterization (ETV + CPC) (RR range 1.16–1.48; high to moderate certainty). For safety, LPS, LPS + LS, and ETV had fewer complications than VPS (RR range 0.20–0.40; high certainty). LPS + LS had fewer complications than LPS (RR 0.49, 95% CrI 0.29–0.79; moderate certainty). Compared with cranial approaches, lumbar surgeries improved favorable outcomes [RR 1.23, 95% confidence interval (CI) 1.19–1.28; moderate certainty], and reduced complications (RR 0.33, 95% CI 0.26–0.43; moderate certainty).

**Conclusion:**

LPS and LPS + LS appeared to be the most efficacious surgical interventions for treating CH, with fewer complications than VPS and ETV + CPC, indicating the potential advantages of lumbar approaches.

**Systematic review registration:**

https://www.crd.york.ac.uk/PROSPERO/view/CRD42024585931, CRD42024585931.

## Graphical abstract

**Figure:**
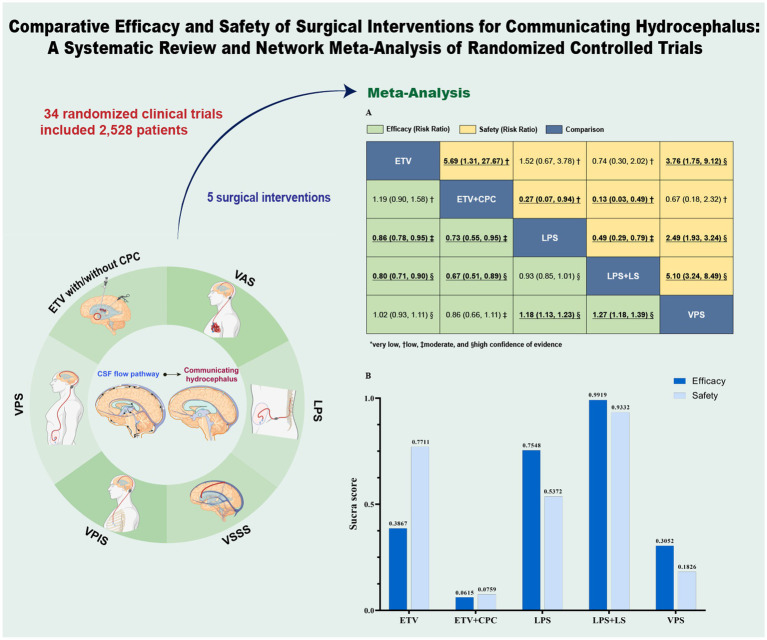

## Introduction

Hydrocephalus is a complex pathological condition characterized by the expansion of the ventricular system due to the abnormal accumulation of CSF (1). As a prevalent neurological disorder, hydrocephalus has a global incidence of approximately 85 cases per 100,000 individuals. Untreated hydrocephalus may lead to brain damage, cognitive impairment, and even death, with a mortality rate ranging 20–87% (2). This condition imposes a significant burden on both patients and society.

Communicating hydrocephalus is a subtype of hydrocephalus caused by impaired CSF absorption that occurs in the absence of CSF flow obstruction (3, 4). Without effective drugs, surgeries remain the primary therapeutic scenarios for CH. The most common surgical approach is the CSF shunt, which includes VPS, ventriculoatrial shunt (VAS), ventriculopleural shunt (VPlS), ventriculosuperior sagittal sinus shunt (VSSS), and LPS. VPS is the standard CSF diversion surgery and has been widely used around the world, especially in America and Europe (5). However, it carries the risk of complications such as intracranial infections, over-drainage, puncture site hematomas, and seizures, which can negatively impact patient outcomes (6, 7). In recent years, LPS has gained popularity, particularly in Asian countries such as Japan and China, where patients often prefer lumbar surgery over cranial procedures (8). Additionally, ETV has shown promise as an alternative treatment for CH, although current evidence on its mechanism remains limited (9).

Although multiple surgical options are available for treating CH, the existing evidence remains sparse, warranting high-quality studies providing references for the optimal choice. This study aims to compare the efficacy and safety of surgeries such as VPS, LPS, and ETV in patients with CH through a systematic review and NMA, and to provide evidence-based recommendations for clinical selection.

## Methods

### Search strategy and selection criteria

This study was conducted in accordance with the Preferred Reporting Items for Systematic Reviews and Meta-Analyses (PRISMA) 2020 statement and the PRISMA Extension Statement for NMA (eMethods in the Supplementary material) (10, 11). The study protocol, including methods and analyses, was prespecified and registered on PROSPERO (CRD 42024585931). To identify all RCTs on CH treatment, we systematically searched PubMed, Embase, the Cochrane Central Register of Controlled Trials, Web of Science, ClinicalTrials.gov, CNKI, Wanfang, Vip, China Biomedical Literature, and the ChiCTR from inception to September 24, 2024. We also searched reference lists of key reviews and meta-analyses to supplement identified citations. Detailed search strategies for each database are provided in the eMethods in the Supplementary material.

**Figure 1:**
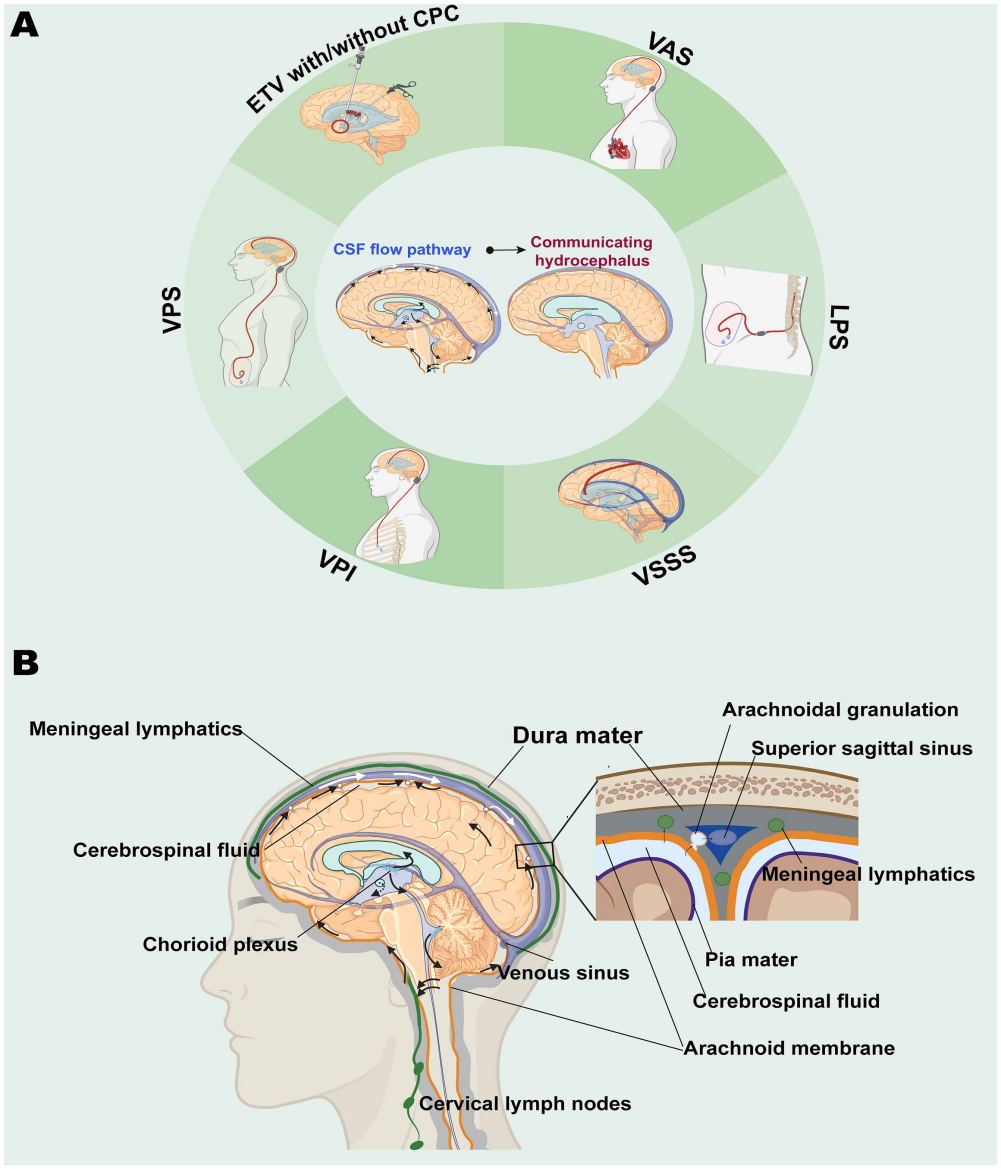
Schematic diagram. **(A)** CH and Its Surgical Interventions. **(B)** Lymphatic pathways of CSF. CSF is primarily produced by the choroid plexus, circulates through the ventricular system into the subarachnoid space, and is absorbed mainly via arachnoid granulations, with additional drainage occurring through extracranial lymphatic pathways. CH results from impaired CSF absorption, leading to progressive ventricular dilation. Surgical treatment options for CH include VPS, VAS, VPlS, VSSS, LPS, and ETV with or without CPC. This figure is created using BioRender (https://biorender.com). Abbreviations: CH, communicating hydrocephalus; CPC, choroid plexus cauterization; CSF, cerebrospinal fluid; ETV, endoscopic third ventriculostomy; LPS, lumboperitoneal shunt; VPS, ventriculoperitoneal shunt; VAS, ventriculopleural shunt; VPlS, ventriculopleural shunt; VSSS, ventriculosuperior sagittal sinus shunt.

For the study design, we included RCTs comparing at least two different surgical methods (Figure 1). The inclusion criteria required patients to have a diagnosis of CH, including hydrocephalus secondary to trauma, hemorrhage, or inflammation, and idiopathic normal pressure hydrocephalus (iNPH). We excluded studies that explicitly included cases of non-CH (obstructive hydrocephalus) or mixed-type hydrocephalus and those that did not clearly specify the type of hydrocephalus.

Two independent researchers selected studies, extracted relevant data, and assessed the risk of bias. The extracted information included the study characteristics (journal name, year of publication, authors, and country of origin), study design, participant characteristics (age, sex, and other demographic details), etiology of hydrocephalus, surgical interventions, study outcomes, follow-up duration and results. For missing data in the original publication, we contacted authors and trial investigators to offer incomplete information. Discrepancies were double-checked and resolved through discussions with other review team members.

### Outcomes

The primary outcomes were efficacy (favorable outcome) and safety (complications) (more details in eMethods in the Supplementary material). Overall, the favorable outcome was defined as improvement in clinical symptoms, requiring no further surgical intervention, with or without reduction of the ventricular system on imaging. Secondary outcomes included revision surgery, infection, seizures, operation time (minutes), and duration of hospitalization (days). Whenever possible, we prioritized the 6 month postoperative time for outcome assessments. Given the extremely poor outcomes of nonsurgical treatments (conservative or delayed surgical interventions) (8), we designated the most widely used VPS as the standard treatment (control group).

### Statistical analysis

Bayesian NMAs were conducted using the GeMTC (version 1.0.2) package in R (version 4.3.2) with Markov Chain Monte Carlo (MCMC) method (12). Uninformative prior distributions were applied for the treatment effects, with a minimally informative prior used for the common standard deviation parameter. Four MCMC chains were set for the initial value, with each chain undergoing 50,000 iterations. To exclude initial value bias, we discarded the first 10,000 annealings and commenced sampling from iteration 10,001. Trace, density and Brooks-Gelman-Rubin diagnosis plots were utilized to visually examine convergence (13). Model fit was evaluated through the posterior total residual deviance and unconstrained data points. Both random-effects (RE) and fixed-effects (FE) models were used to pool network results, with model selection regarding deviance information criterion (DIC) (14). Details of the Bayesian model specifications were provided in eMethods.

Network graphs were scaled based on the number of studies and patients for each treatment node. Summary RRs were estimated for dichotomous outcomes and mean differences (MDs) for continuous outcomes, with 95% CrIs in the NMAs. League tables, two-dimensional graphs, and forest plots were used to visualize relative treatment effects in network estimations. Treatment rank probabilities were calculated, and SUCRA values were generated to display cumulative ranking probability plots for the interventions. Higher SUCRA values indicate better intervention effects.

Following the Cochrane Handbook for Systematic Reviews, statistical heterogeneity in NMA and each pairwise comparison was assessed using *I*^2^ statistics and visual inspection of forest plots (15). We appraised the clinical and methodological characteristics of the included studies to determine the appropriateness of the transitivity assumption. Inconsistencies between direct and indirect evidence were assessed using global and local approaches. Global inconsistency was assessed by comparing residual deviance and DIC between the unrelated study effects and consistent models (14). Local inconsistency was assessed using the node-splitting approach where relevant head-to-head trials were available (16). Small-study effects and publication bias were assessed using funnel plots if at least 10 studies were available (17). Conventional pairwise meta-analyses were conducted for comparisons involving head-to-head studies. To compare the overall efficacy and safety of lumbar versus cranial surgical interventions, we consolidated LPS and LPS + LS into the unified lumbar approach, while merging VPS and ETV with or without CPC into the cranial approach. Statistical significance was set at *p* < 0.05, and all *p*-values two-tailed.

We assessed the risk of bias in individual studies using the Cochrane Risk of Bias tool for Randomized trials version 2 (RoB2) (18). The certainty of evidence was evaluated using the Confidence in Network Meta-Analysis (CINeMA) framework (19) and Grading of Recommendations, Assessment, Development, and Evaluation (GRADE) (20, 21).

To assess potential heterogeneity in treatment effects, we performed several sensitivity analyses stratified by: (1) age: pediatric patients (age <18 years) versus adults (age ≥18 years); (2) statistical model selection: fitting both RE and FE models; (3) etiology: reporting outcomes separately for post-hemorrhagic hydrocephalus, post-infectious hydrocephalus, and iNPH; (4) evidence quality: excluding studies with a high risk of bias; (5) follow-up duration: excluding studies with less than 3 months of follow-up.

## Results

### Study selection and characteristics

**Figure 2:**
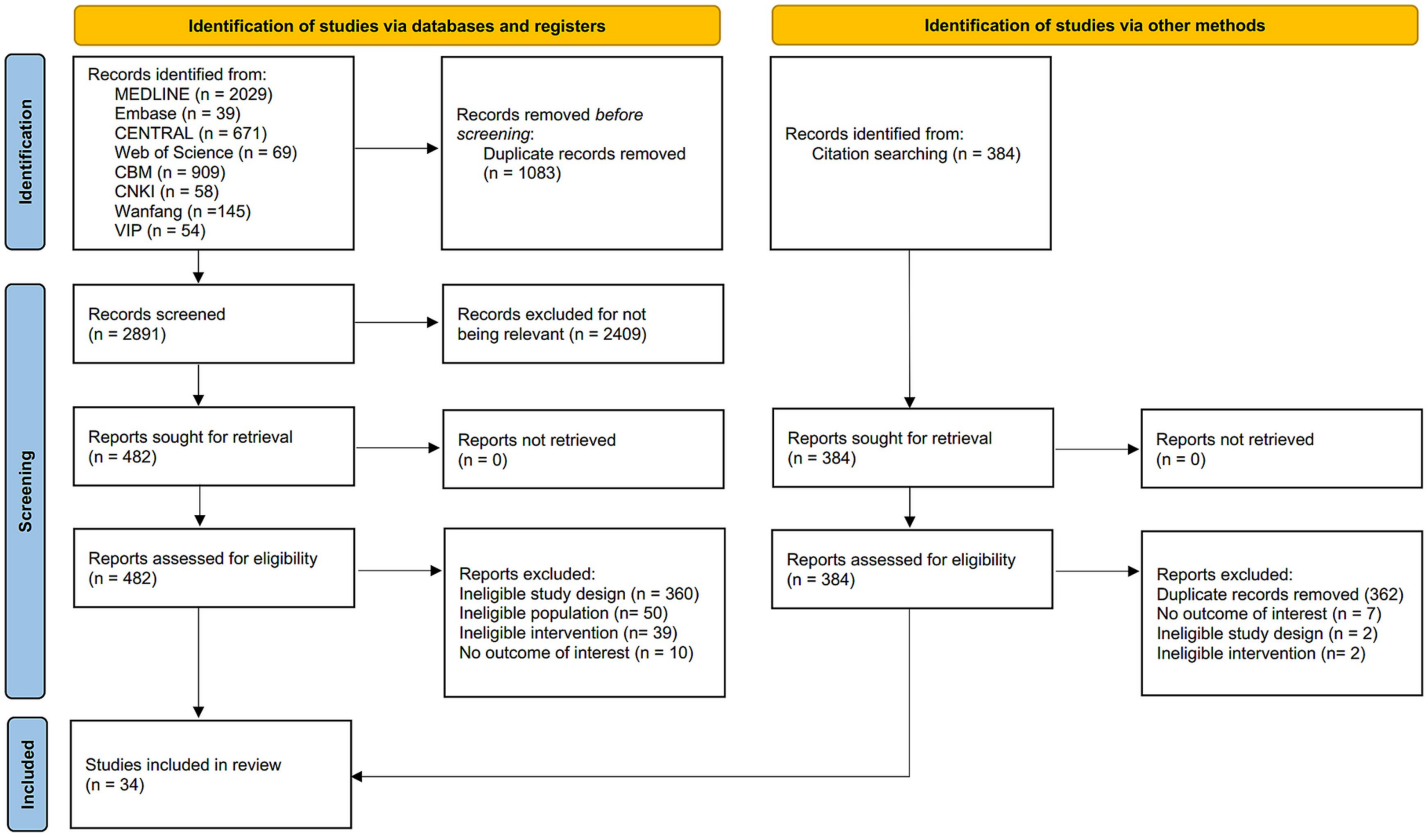
PRISMA flow diagram.

We identified 4,358 citations through database searches and relevant reviews, with 1,445 duplicates removed. After screening 2,913 titles and abstracts and 504 full texts, a total of 34 RCTs involving five surgical interventions between 2013 and 2024 were included in this NMA (Figure 2) (22–55).

The 34 eligible studies included 2,528 participants (33–300 per trial). Study characteristics are in Table 1. Among the 34 trials, the mean age ranged from 3.1 months to 71 years (for trials that reported mean or median age), with six studies (17.6%) exclusively focusing on the pediatric population. The proportion of male patients ranged from 50.0–76.7%. Most of the trials (*n* = 32, 94.1%) recruited patients from Asia. A single trial (2.9%) examined three treatment groups. The interventions included VPS, ETV, LPS, LPS with laparoscope (LPS + LS), and ETV + CPC. The risk of bias assessment for eligible trials for each outcome is provided in Supplementary Table 1. Twenty-five (73.5%) trials were judged as having a low risk of bias, eight (23.5%) as having some concern, and one (2.9%) as having a high risk of bias due to missing outcome data.

**Table 1:** Characteristics of included studies.

Study ID	Country	Intervention and sample size	Age	Male num (%)	Hydrocephalus etiology/classification	Duration of follow-up	Outcomes^a^
Kulkarni et al. (49)	Uganda	ETV + CPC: 51 vs. VPS: 49	3.1 m vs. 3.1 m	60.8% vs. 63.3%	Post-infectious hydrocephalus	12 m	A, B, C, D
Punchak 2019 (51)	Uganda	ETV + CPC: 51 vs. VPS: 49	3.25 m	NA	Post-infectious hydrocephalus	24 m	E
Goyal et al. (24)	India	ETV: 24 vs. VPS: 24	4.21 y vs. 4.31 y	70.8% vs. 70.8%	Post-infectious hydrocephalus	6 m	A, C, D
Pinto 2013 (50)	Brazil	ETV: 16 vs. VPS: 26	71 y vs. 70 y	NA	iNPH	12 m	A
Huang et al. (36)	China	LPS: 15 vs. VPS: 15 vs. LPS + LS: 15	46.8 ± 11.6 y vs. 44.9 ± 10.5 y vs. 43.2 ± 12.3 y	60% vs. 53.3% vs. 53.3%	CH	6 m	A, B, C
Zhang et al. (34)	China	ETV: 21 vs. VPS: 21	6.81 ± 2.43 m vs. 7.25 ± 2.58 m	61.9% vs. 52.4%	CH	8 m	A, C
Han 2016 (54)	China	LPS + LS: 36 vs. LPS: 36	46.38 ± 12.05 y vs. 47.19 ± 12.23 y	61.1% vs. 55.6%	CH	6 m	A, B, C
Gong et al. (35)	China	LPS + LS: 30 vs. LPS: 30	43.2 y vs. 42.4 y	70% vs. 76.7%	CH	6 m	A, C, D, F
Chen (37)	China	LPS: 45 vs. VPS: 45	45.69 ± 10.22 y vs. 45.10 ± 10.47 y	53.3% vs. 57.8%	CH	6 m	A, B, C, E
Wu et al. (30)	China	LPS: 150 vs. VPS: 150	52.15 ± 9.28 y vs. 52.66 ± 9.41 y	58.7% vs. 54%	CH	6 m	A, D
Li et al. (44)	China	VPS: 45 vs. LPS + LS: 45	36.15 ± 10.26 y vs. 36.67 ± 10.49 y	53.3% vs. 51.1%	CH	6 m	A, B, C, D
Wang (45)	China	LPS: 46 vs. VPS: 46	50.17 ± 6.82 y vs. 49.68 ± 6.35 y	63% vs. 60.9%	CH	6 m	A, B, C, E, F, G
Liang (41)	China	LPS: 21 vs. VPS: 21	43.64 ± 4.72 y vs. 42.58 ± 4.38 y	61.9% vs. 57.1%	CH	6 m	A, B, C
Wang et al. (38)	China	LPS: 34 vs. VPS: 34	44.89 ± 7.44 y vs. 44.56 ± 7.37 y	58.8% vs. 52.9%	CH	1 m	A, B, C, E, F, G
Li 2017 (55)	China	LPS: 31 vs. VPS: 31	42.19 ± 3.22 y vs. 42.85 ± 3.48 y	64.5% vs. 67.7%	CH	6 m	A, B, C
Liu and Zhang (32)	China	VPS: 61 vs. LPS: 61	48.32 ± 3.65 y vs. 48.46 ± 3.58 y	52.5% vs. 54.1%	Post-hemorrhagic hydrocephalus	1 m	A, B, C, F, G
Guo et al. (26)	China	VPS: 38 vs. LPS + LS: 38	35.79 ± 9.41 y vs. 35.06 ± 9.32 y	50% vs. 55.3%	CH	8 m	A, B, C
Du and Sun (25)	China	LPS: 30 vs. VPS: 30	35.45 ± 3.85 y vs. 35.26 ± 2.45 y	50% vs. 53.3%	CH	6 m	A, B, C
Huang et al. (46)	China	LPS: 15 vs. VPS: 15	34.1 ± 2.3 y	NA	CH	6 m	A, B, C
Ye (47)	China	LPS: 25 vs. VPS: 25	54.45 ± 6.26 y vs. 53.39 ± 6.53 y	60% vs. 52%	CH	1 w	A, B, C, E
Lu et al. (42)	China	VPS: 68 vs. LPS + LS: 62	43.1 ± 6.8 y vs. 42.5 ± 7.0 y	58.8% vs. 59.7%	CH	6 m	A, D, B, C, E
Wu et al. (27)	China	LPS: 46 vs. VPS: 46	45.32 ± 6.38 y vs. 45.37 ± 6.4 y	63% vs. 58.7%	CH	6 m	A, B, C, E
Li 2023 (53)	China	ETV: 36 vs. VPS: 46	43.65 ± 2.24 y vs. 45.08 ± 3.98 y	69.4% vs. 56.5%	CH	3 m	A, B, C
Zang and Wang (43)	China	ETV: 40 vs. VPS: 40	17.5 ± 2.3 m vs. 17.7 ± 2.6 m	60% vs. 62.5%	CH	1 m	A, B, C, D, F, G
Chen et al. (33)	China	LPS: 40 vs. VPS: 40	46.70 ± 10.23 y vs. 48.21 ± 11.24 y	70% vs. 72.5%	CH	1 w	A, B, C
Xiong and Ai (31)	China	VPS: 39 vs. LPS: 39	45.5 ± 1.0 y vs. 45.0 ± 1.0 y	74.4% vs. 71.8%	CH	1 m	C, F, G
Raut et al. (22)	Pakistan	VP: 30 vs. ETV: 30	35.1 ± 9.9 y vs. 35.0 ± 8.5 y	50% vs. 66.7%	Post-infectious hydrocephalus	1 m	A
Aranha et al. (23)	India	ETV: 15 vs. VPS: 18	<18 y	NA	Post-infectious hydrocephalus	5 m	A
Li and Lu (29)	China	VPS: 30 vs. LPS: 30	42.22 ± 6.94 y vs. 40.34 ± 6.48 y	66.7% vs. 60%	CH	12 m	A, B, C, F, G
Zhang 2018 (52)	China	VPS: 21 vs. LPS: 21	40.0 ± 25.5 y vs. 40.5 ± 25.5 y	52.4% vs. 47.6%	CH	12 m	A
Hu et al. (39)	China	LPS: 25 vs. VPS: 25	37.3 ± 8.9 y vs. 37.6 ± 9.2 y	52% vs. 56%	CH	1 m	A, C, F, G
Liu and Xiao (40)	China	LPS: 35 vs. VPS: 35	56.62 ± 6.33 y vs. 56.09 ± 6.29 y	51.4% vs. 54.3%	CH	1 w	B
Cheng et al. (48)	China	LPS: 25 vs. VPS: 25	49.5 ± 10.6 y vs. 49.9 ± 10.1 y	56% vs. 56%	Post-hemorrhagic hydrocephalus	1 w	A, B, C, E
Su et al. (28)	China	ETV: 70 vs. VPS: 70	68.13 ± 7.24 y vs. 65.33 ± 4.94 y	61.4% vs. 52.9%	Post-hemorrhagic hydrocephalus	8 m	A, B, C, F, G

a A, Favorable outcome; B, Complications; C, Infection; D, Revision; E, Seizure; F, Operation time; G, Duration of hospitalization.

CH, Communicating hydrocephalus; ETV, Endoscopic third ventriculostomy; ETV + CPC, Endoscopic third ventriculostomy with choroid plexus cauterization; iNPH, idiopathic normal pressure hydrocephalus; LPS, Lumboperitoneal shunt; LPS + LS, Lumboperitoneal shunt with laparoscope; NA, Not applicable due to no available data; VPS, Ventriculoperitoneal shunt.

### Synthesis of results

**Figure 3:**
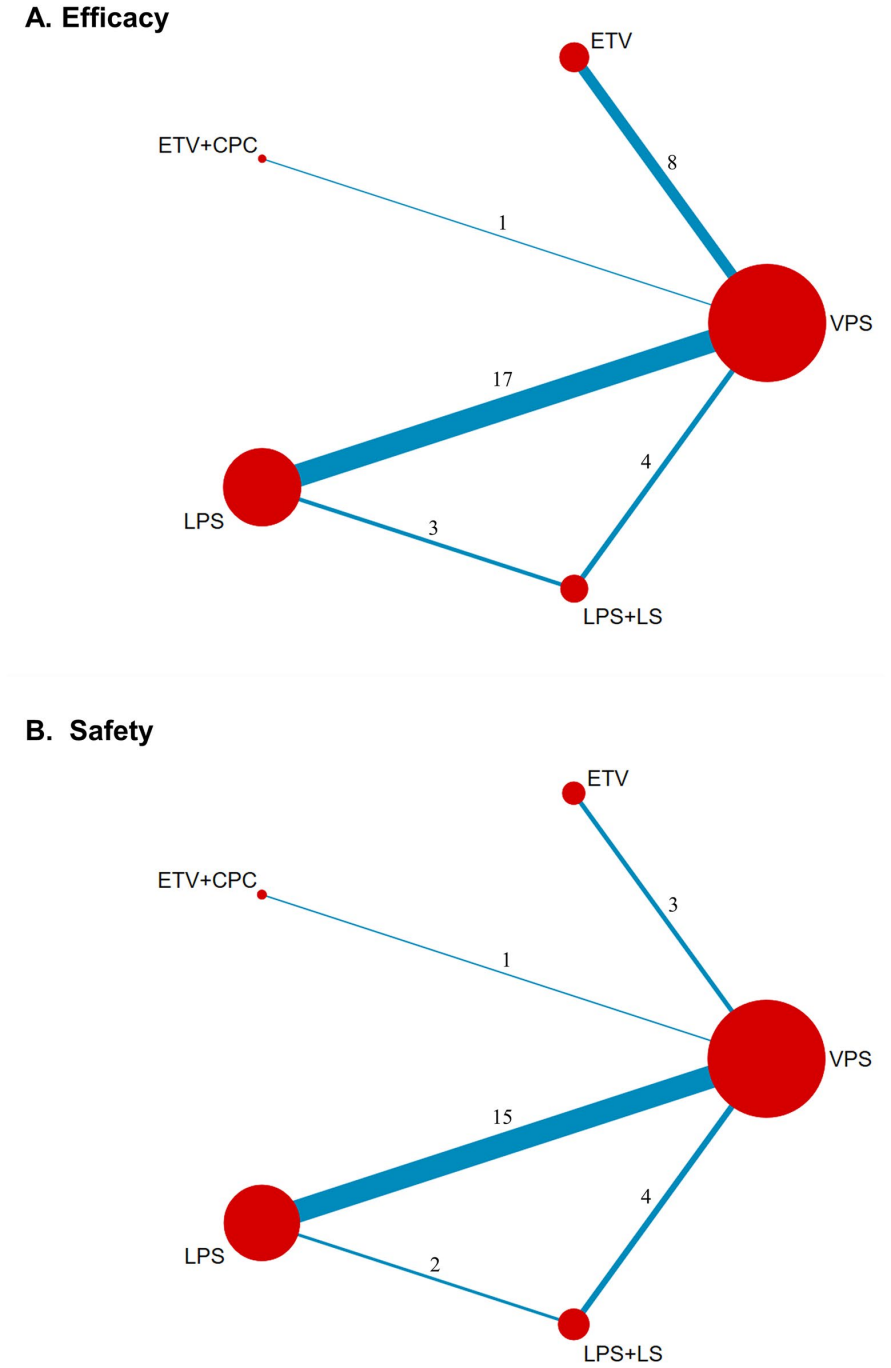
NMA of eligible comparisons for efficacy **(A)** and safety **(B)**. The node size is proportional to the number of participants in each surgical intervention. The widths of the lines linking the treatments represent the number of trials with direct comparisons.

A network of eligible comparisons for the primary and secondary outcomes is presented in Figure 3 and Supplementary Figure 1. The geometry of the evidence network indicated that all surgical interventions were evaluated in at least one RCT, with most comparisons involving VPS.

**Figure 4:**
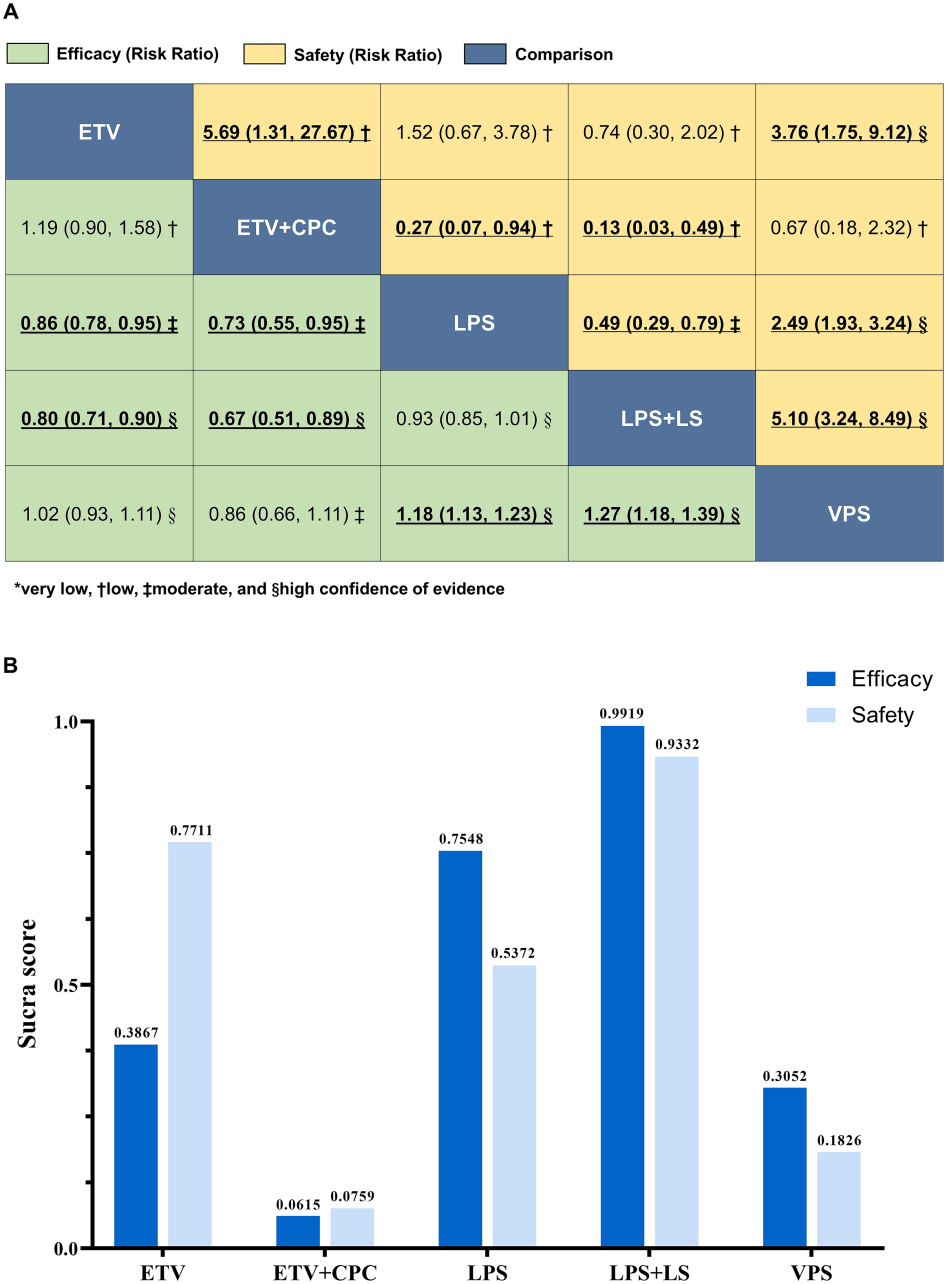
Relative effects of surgical interventions for primary outcomes. **(A)** League tables of NMA for efficacy (green) and safety (yellow). Data are presented as RRs (95% CrIs), with the column-defining treatment compared against the row-defining treatment. Statistically significant results are **bolded** and underscored. The certainty of the evidence was classified according to the CINeMA framework as *very low, †low, ‡moderate, and §high. Treatments are listed alphabetically in all tables. **(B)** SUCRA scores for efficacy (favorable outcome) and safety (complications). Higher SUCRA values indicated better intervention effects. CrIs, Credible intervals; ETV, Endoscopic third ventriculostomy; ETV + CPC, Endoscopic third ventriculostomy with choroid plexus cauterization; LPS, Lumboperitoneal shunt; LPS + LS, Lumboperitoneal shunt with laparoscope; RRs, Risk ratios; SUCRA, Surface under the Cumulative Ranking Curve; VPS, Ventriculoperitoneal shunt.

Figure 4 presents the NMA league table and SUCRA scores for the primary outcome. To ensure the certainty of the evidence, we incorporated CINeMA assessments into the league table. The quality of evidence graded by CINeMA ranged from low to high, with low-quality evidence primarily attributed to major concerns in imprecision and incoherence (Supplementary Table 2). We found no evidence of funnel plot asymmetry (Supplementary Figure 2). The results of the model fit statistics for the FE consistency, RE consistency, and inconsistency models are presented in Supplementary Table 3. FE models were selected for most outcomes except operation time and duration of hospitalization. The leverage plots are provided in Supplementary Figure 3. No evidence of global inconsistency was found (Supplementary Table 3). However, node-splitting analysis revealed some local inconsistencies in complications and seizures (Supplementary Figure 4). We verified the data for errors, violations of homogeneity or transitivity, and other sources of inconsistency. No errors were found in data extraction or statistics, and no significant variables differed across comparisons.

### Primary outcomes

In terms of favorable outcome (31 RCTs, 2,390 patients), LPS (RR 1.18, 95% CrI 1.13–1.23, high certainty) and LPS + LS (RR 1.27, 95% CrI 1.18–1.39, high certainty) were more effective compared to VPS (Figure 4A). Additionally, both LPS and LPS + LS outperformed ETV with or without CPC (RR range 1.16–1.48; high to moderate certainty). Figure 4B and Supplementary Figure 5 present the SUCRA curve score, ranking each surgical intervention. LPS + LS ranked highest (SUCRA 99.2%), followed by LPS (75.5%), ETV (38.7%), and VPS (30.5%), with ETV + CPC having the lowest score (6.2%).

In the NMA of complications (23 RCTs, 1,783 patients), LPS, LPS + LS, and ETV significantly reduced adverse events compared to VPS, with RR ranging from 0.20–0.40, supported by high-certainty evidence (Figure 4A). LPS + LS had fewer complications than LPS (RR 0.49, 95% CrI 0.29–0.79; moderate certainty). The SUCRA curve (Figure 4B; Supplementary Figure 5) suggests that LPS + LS has the lowest risk of adverse events (SUCRA 93.3%), followed by ETV (77.1%) and LPS (53.7%), while ETV + CPC has the highest risk (7.6%).

### Pooled analysis

**Figure 5:**
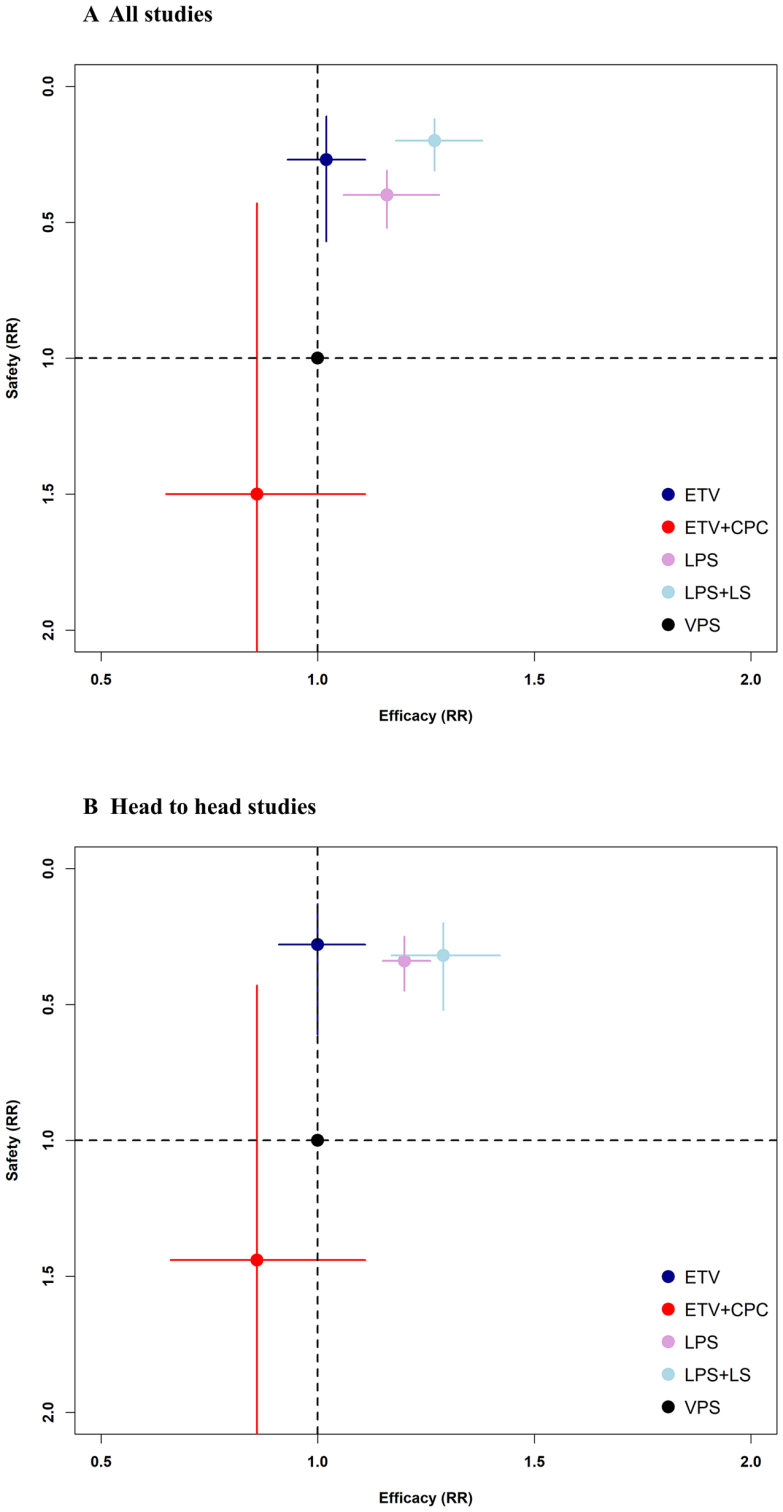
Two-dimensional graphs about efficacy and safety in all studies **(A)** and head-to-head studies only **(B)**. Data are reported as RRs in comparison with VPS (reference treatment). Error bars indicate 95% CIs. Individual surgeries are represented by differently colored nodes. ETV, Endoscopic third ventriculostomy; ETV + CPC, Endoscopic third ventriculostomy with choroid plexus cauterization; LPS, Lumboperitoneal shunt; LPS + LS, Lumboperitoneal shunt with laparoscope; RRs, Risk ratios; VPS, Ventriculoperitoneal shunt.

Figure 5 shows two-dimensional graphs of efficacy versus safety in all studies and head-to-head studies. Overall, both analyses yielded similar results. In all studies, LPS + LS was the optimal surgical intervention for efficacy and safety. In head-to-head comparisons, LPS and LPS + LS were superior to VPS in both efficacy (LPS: RR 1.20, 95% CI 1.15–1.26; LPS + LS: RR 1.29, 95% CI 1.17–1.42) and safety (LPS: RR 0.34, 95% CI 0.25–0.45; LPS + LS: RR 0.32, 95% CI 0.20–0.52). ETV was superior to VPS only in safety (RR 0.28, 95% CI 0.13–0.61) (Supplementary Figure 6).

**Figure 6:**
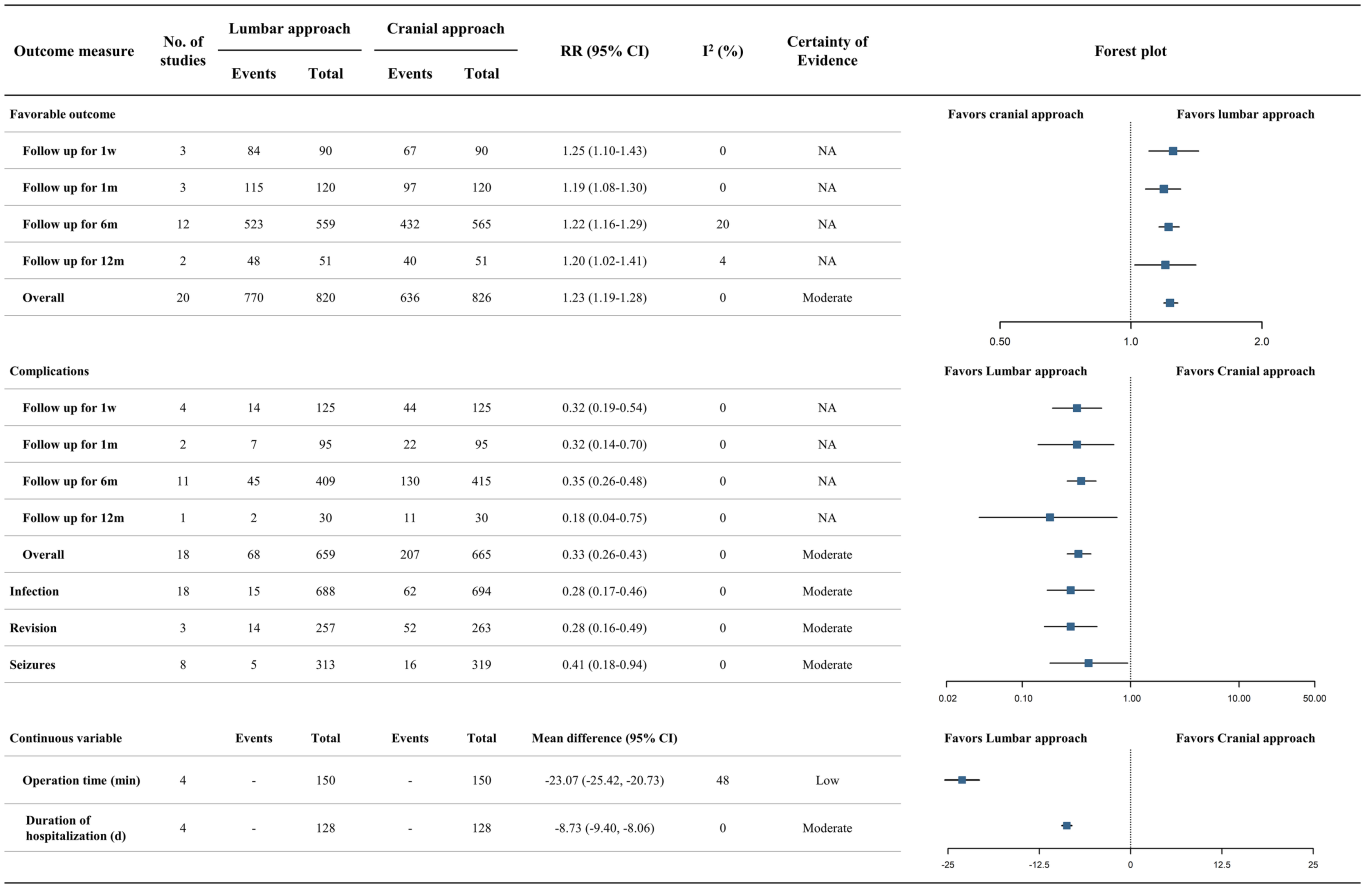
Forest plot of lumbar approach versus cranial approach. The forest plot shows the outcomes of lumbar approach versus cranial approach for treating CH at 1 week, 1 month, 6 months, and 12 months. Summary RRs were estimated for dichotomous outcomes, while MDs were used for continuous outcomes, both with 95% CIs. The certainty of evidence was evaluated by the GRADE.

The pooled analysis comparing lumbar and cranial approaches was shown in Figure 6, with certainty of evidence assessed using the GRADE approach (Supplementary Table 4). Due to the absence of head-to-head comparisons between LPS or LPS + LS and ETV or ETV + CPC, the cranial approach in our analysis effectively comprised only VPS. Across various follow-up time points from 1 week to 12 months, lumbar surgeries were superior to cranial surgeries in both efficacy (RR 1.23, 95% CI 1.19–1.28; moderate certainty) and safety (RR 0.33, 95% CI 0.26–0.43; moderate certainty) (Figure 6; Supplementary Table 4). Moreover, compared with cranial access, lumbar approach significantly reduced the incidence of infection (RR 0.28, 95% CI 0.17–0.46), revision (RR 0.28, 95% CI 0.16–0.49), and seizures (RR 0.41, 95% CI 0.18–0.94), and significantly shortened both operative time (MD −23.07, 95% CI −25.42, −20.73) and duration of hospitalization (MD −8.73, 95% CI −9.40, −8.06) (Figure 6).

### Secondary outcomes

Results of secondary outcomes are presented in Supplementary Figures 5, 7. For infection (27 RCTs, 1,991 patients), ETV, LPS, and LPS + LS significantly reduced infection rates compared to VPS, with RR ranging from 0.06–0.29, supported by moderate-to-high certainty of evidence. In seven RCTs (808 patients), LPS and LPS + LS significantly reduced the risk of revision compared to VPS, ETV, and ETV + CPC (LPS: RR 0.27–0.39; LPS + LS: RR 0.10–0.37, moderate certainty). In nine trials (717 patients), only LPS significantly reduced seizure incidence compared to VPS (RR 0.29, 95% CrI 0.09–0.81, moderate certainty). In nine trials (750 patients), the operation time for LPS, LPS + LS, and ETV were significantly shorter than for VPS, with MD ranging from −19.78 to −34.77 (moderate evidence). In eight RCTs (690 patients), compared to VPS, both ETV (MD −4.41, 95% CrI −8.55 to −0.29, moderate evidence) and LPS (MD −7.23, 95% CrI −9.74 to −4.79, moderate evidence) significantly shortened the duration of hospitalization.

Subgroup and sensitivity analyses based on age, statistical model selection, etiology, evidence quality, and follow-up duration did not reveal substantial variation in the results (Supplementary Figure 8). For infant CH, no RCTs evaluated LPS or LPS + LS. Among the available interventions, VPS ranked highest in both efficacy and safety, followed by ETV and ETV + CPC. For adult CH, LPS + LS demonstrated the highest efficacy, followed by LPS, ETV, and VPS. In safety, LPS + LS also ranked first, with ETV, LPS, and VPS following in descending order. No RCTs evaluating ETV + CPC were identified for adult CH. For iNPH, LPS + LS was again the most efficacious intervention, followed by LPS, VPS, and ETV, though no RCTs reported comparative safety outcomes for this specific population (Supplementary Figure 8).

## Discussion

This systematic review and NMA of RCTs found high-to-moderate certainty evidence supporting LPS and LPS + LS as the most efficacious surgical options for favorable outcome compared to VPS and ETV with or without CPC. High certainty evidence indicated that LPS, LPS + LS, and ETV had the best safety profiles, with fewer complications than VPS. Lumbar approaches demonstrated superior efficacy and safety over cranial surgery, reducing infection, reoperation, seizures, operative time and hospital stay. Subgroup and sensitivity analyses confirmed the robustness of the results.

Hydrocephalus arises from diverse pathologies affecting the complex CSF circulating system (1). Addressing hydrocephalus by targeting its underlying causes, rather than focusing on ventricular enlargement, is essential for achieving meaningful improvements for patients. Currently, no guidelines provide a clear choice or ranking of surgical treatments for CH. CH has traditionally been treated with CSF shunting, based on the theory that CH results from elevated CSF outflow resistance, which the shunt normalizes by offering an alternative CSF pathway (Figure 1) (9).

Despite VPS remains the most commonly employed method in North America and Europe, it is associated with complications including intracerebral hematoma, subdural effusion, and infection (6, 7). VPS is also unsuitable for patients at risk of seizures or other relevant contraindications (5). LPS, through the lumbo-peritoneal approach, avoids cranial surgery, reducing complications from ventricular shunting and making it a viable option for patients with a preexisting seizure disorder.

A meta-analysis of 25 studies found LPS as safe and effective as VPS for treating CH (7). Our study demonstrated that LPS significantly improved outcome and reduced adverse events compared to VPS. LPS also significantly reduced seizure risk compared to VPS, a key advantage of lumbar surgery over cranial surgery. Two meta-analyses reported comparable outcomes and adverse event rates across VPS, VAS, LPS, and ETV for iNPH (51, 52). However, both prior studies adopted proportional meta-analytical approaches, in which outcomes were synthesized as pooled event rates for each intervention considered in isolation. This methodology is useful for estimating the absolute performance of individual procedures but does not allow formal comparisons between interventions, nor does it account for the relative effects of competing surgical strategies (53). By contrast, our NMA is a comparative framework that integrates evidence from both direct head-to-head trials and indirect comparisons across a connected network of interventions, thereby enabling estimation of relative treatment effects and probabilistic ranking of multiple options within a single coherent model (15). Methodological differences and variations in study inclusion criteria may account for the discrepancies in findings.

Laparoscopy-assisted peritoneal access in VPS and LPS placement offers benefits, particularly for patients with abdominal obesity (54, 55). Our findings are consistent with these reports, showing that laparoscopic-assisted LPS further enhances efficacy and safety, making it a potentially optimal surgical approach for CH.

ETV, an option mainly for obstructive hydrocephalus, has also been explored for CH recently (56–58). ETV can bypass the aqueduct of Sylvius, the outlet foramina of the fourth ventricle, and obstructions at the basal cisterns. Blockage between the spinal and cortical subarachnoid spaces may explain the successful management of intraventricular NPH with ETV (1, 57). However, the physiological basis for ETV in treating CH remains unclear, and the procedure is generally considered inappropriate or less effective for this subtype (59). In our study, ETV showed no significant difference in efficacy compared to VPS, despite its lower complication rate, which may be attributed to the avoidance of shunt-related adverse events. Notably, with limited medical resources, ETV may be a more sustainable and potentially cost-effective option, where avoidance of shunt-related complications, lifelong follow-up, and repeated surgical interventions may reduce long-term economic burden (60, 61).

In addition, we found that combining ETV with CPC significantly worsened the safety profile. The inferior safety profile of ETV + CPC may reflect the higher technical complexity of performing both procedures simultaneously, particularly in very young infants, as well as the influence of the surgical learning curve and variability in the extent of CPC (62). Patient selection also appears important, with younger age and certain etiologies (e.g., post-hemorrhagic or post-infectious hydrocephalus) associated with lower success and higher early failure (63). Overall, current evidence on ETV + CPC safety remains limited, underscoring the need for further high-quality studies (64). Furthermore, although existing studies from Africa, such as the seminal work by Warf (65), have primarily demonstrated the efficacy of ETV + CPC in reducing shunt dependency. However, longitudinal data on long-term outcomes—such as cognitive function, endocrine-metabolic status, or brain volumetrics as patients transition into adolescence and adulthood—have not been systematically reported. From a theoretical perspective, concerns have been raised that sustained reduction in CSF production may have unknown effects on long-term brain homeostasis, waste clearance, and neurodevelopment, particularly in pediatric patients who will live with these alterations for decades. These uncertainties underscore the need for cautious interpretation of ETV + CPC outcomes and highlight the importance of long-term, prospective studies with detailed neurodevelopmental follow-up before broad generalization of this approach.

### Limitations

Our study had some limitations. First, the lack of a standardized grading system for hydrocephalus led to imprecise outcome definitions. However, we detailed the definitions of favorable outcomes in each original study (eMethods in the Supplementary material) and synthesized them based on expert consensus. The *I*^2^ statistics indicated no statistical heterogeneity. Second, this same lack of a standardized grading system also limited the precision of severity assessment across studies. Consequently, subgroup analyses based on severity indices (such as Evans’ index or clinical severity scores) could not be performed. These limitations constrain the direct clinical applicability of our findings. Therefore, while our analysis provides a robust evidence base, the conclusion suggesting the superiority of lumbar approaches should be interpreted with caution in clinical practice, particularly in severe cases with significantly elevated intracranial pressure or marked ventricular dilation. Third, the included studies originated from Asia, Africa, and South America, with the majority from Asia. This geographic distribution did not result from the deliberate exclusion of trials conducted in North America or Europe. Rather, it reflects the current global research landscape in this specific field. To ensure a rigorous comparison of surgical strategies for CH, we restricted inclusion to RCTs that (1) employed random allocation, (2) directly compared different surgical procedures, and (3) enrolled patients with a clearly defined diagnosis of CH. Based on comprehensive and systematic searches across multiple major databases, no trials from North America or Europe were identified. Given known regional differences in surgical practice patterns, healthcare systems, and patient characteristics, caution is warranted when extrapolating these findings to Western populations, and further high-quality RCTs from diverse geographic regions are needed to enhance external validity. Fourth, including both adult and pediatric CH may have influenced the transitivity assumption. To address this concern, we performed prespecified subgroup analyses for adults and infants, and the results remained unchanged. Fourth, data on several less common surgeries including VAS, VPlS, VSSS, and laparoscopy-assisted VPS, were unavailable due to the lack of eligible RCTs. However, our study covers current primary clinical treatment scenarios for CH. Fifth, CINeMA assessed some comparisons as low or very low quality. Censored adverse event data below a pre-specified study-dependent threshold may bias the estimated incidence in these comparisons. We integrated the certainty of evidence into our results and interpreted them with caution. Future RCTs should place greater emphasis on censored adverse event including secondary tonsillar descent or Chiari formation to provide more comprehensive evidence.

## Conclusion

This NMA provides the most comprehensive evidence for decision-making on CH management. Comparisons of surgical options should be interpreted with caution due to current evidence limitations, patients’ specific contraindications, and regional variations in clinical practice. LPS and LPS + LS appeared to be the most efficacious surgical interventions for treating CH, with fewer complications than VPS and ETV + CPC, indicating the potential advantages of lumbar surgeries. Future studies are warranted to generate high-quality evidence from direct comparisons between lumbar approaches and ETV with or without CPC.

## Data Availability

The original contributions presented in the study are included in the article/Supplementary material, further inquiries can be directed to the corresponding authors.
