# Supplementary material for: Comparative efficacy and safety of surgical interventions for communicating hydrocephalus: a systematic review and network meta-analysis of randomized controlled trials
Source: Front Neurol. 2026 Feb 25;17:1763131. doi: 10.3389/fneur.2026.1763131 (PMC12977094; doi:10.3389/fneur.2026.1763131)
Supplement: Supplementary file 1 [file Supplementary_file_1.docx]

**Supplementary Online Content**

**eMethods**

1. PRISMA NMA Checklist.

2. Search Strategy for Databases.

3. Definition of Outcomes.

4. Details of the Bayesian model specifications

**eTable 1.** Risk of Bias Assessment.

**eTable 2.** CINeMA Assessment.

**eTable 3.** Assessment of Network Model Fit.

**eTable 4.** GRADE Assessment.

**eFigure 1.** Network plot for Secondary Outcomes.

**eFigure 2.** Funnel Plots.

**eFigure 3.** Leverage plot of Consistency Models for Network Meta-Analysis by Outcomes.

**eFigure 4.** Inconsistency Analysis Using the Node-splitting Approach.

**eFigure 5.** SUCRA ranking for Primary and Secondary Outcomes.

**eFigure 6.** Conventional Pairwise Meta-analyses across Comparisons.

**eFigure 7.** League plot for Secondary Outcomes.

**eFigure 8.** Sensitivity Analyses for the Network Meta-Analysis of Primary Outcomes.

**eMethods**

**1. PRISMA NMA checklist**

**PRISMA NMA Checklist of Items to Include When Reporting A Systematic Review Involving a Network Meta-analysis**

| **Section/Topic** | **Item #** | **Checklist Item** | **Reported on Page #** |
| --- | --- | --- | --- |
| **TITLE** |  |  |  |
| Title | 1 | Identify the report as a systematic review *incorporating a network meta-analysis (or related form of meta-analysis).* | Title |
|  |  |  |  |
| **ABSTRACT** |  |  |  |
| Structured summary | 2 | Provide a structured summary including, as applicable:  **Background:** main objectives  **Methods:** data sources; study eligibility criteria, participants, and interventions; study appraisal; and *synthesis methods, such as network meta-analysis.*  **Results:** number of studies and participants identified; summary estimates with corresponding confidence/credible intervals; *treatment rankings may also be discussed. Authors may choose to summarize pairwise comparisons against a chosen treatment included in their analyses for brevity.*  **Discussion/Conclusions:** limitations; conclusions and implications of findings.  **Other:** primary source of funding; systematic review registration number with registry name. | Abstract |
|  |  |  |  |
| **INTRODUCTION** |  |  |  |
| Rationale | 3 | Describe the rationale for the review in the context of what is already known*, including mention of why a network meta-analysis has been conducted.* | Introduction |
| Objectives | 4 | Provide an explicit statement of questions being addressed, with reference to participants, interventions, comparisons, outcomes, and study design (PICOS). | Introduction |
|  |  |  |  |
| **METHODS** |  |  |  |
| Protocol and registration | 5 | Indicate whether a review protocol exists and if and where it can be accessed (e.g., Web address); and, if available, provide registration information, including registration number. | Search Strategy and Selection Criteria |
| Eligibility criteria | 6 | Specify study characteristics (e.g., PICOS, length of follow-up) and report characteristics (e.g., years considered, language, publication status) used as criteria for eligibility, giving rationale. *Clearly describe eligible treatments included in the treatment network, and note whether any have been clustered or merged into the same node (with justification).* | Search Strategy and Selection Criteria |
| Information sources | 7 | Describe all information sources (e.g., databases with dates of coverage, contact with study authors to identify additional studies) in the search and date last searched. | Search Strategy and Selection Criteria |
| Search | 8 | Present full electronic search strategy for at least one database, including any limits used, such that it could be repeated. | Search Strategy and Selection Criteria |
| Study selection | 9 | State the process for selecting studies (i.e., screening, eligibility, included in systematic review, and, if applicable, included in the meta-analysis). | Search Strategy and Selection Criteria |
| Data collection process | 10 | Describe method of data extraction from reports (e.g., piloted forms, independently, in duplicate) and any processes for obtaining and confirming data from investigators. | Search Strategy and Selection Criteria |
| Data items | 11 | List and define all variables for which data were sought (e.g., PICOS, funding sources) and any assumptions and simplifications made. | Outcomes |
| **Geometry of the network** | **S1** | Describe methods used to explore the geometry of the treatment network under study and potential biases related to it. This should include how the evidence base has been graphically summarized for presentation, and what characteristics were compiled and used to describe the evidence base to readers. | Statistical Analysis |
| Risk of bias within individual studies | 12 | Describe methods used for assessing risk of bias of individual studies (including specification of whether this was done at the study or outcome level), and how this information is to be used in any data synthesis. | Statistical Analysis |
| Summary measures | 13 | State the principal summary measures (e.g., risk ratio, difference in means). *Also describe the use of additional summary measures assessed, such as treatment rankings and surface under the cumulative ranking curve (SUCRA) values, as well as modified approaches used to present summary findings from meta-analyses.* | Statistical Analysis |
| Planned methods of analysis | 14 | Describe the methods of handling data and combining results of studies for each network meta-analysis. This should include, but not be limited to:   - *Handling of multi-arm trials;* - *Selection of variance structure;* - *Selection of prior distributions in Bayesian analyses; and* - *Assessment of model fit.* | Statistical Analysis |
| **Assessment of Inconsistency** | **S2** | Describe the statistical methods used to evaluate the agreement of direct and indirect evidence in the treatment network(s) studied. Describe efforts taken to address its presence when found. | Statistical Analysis |
| Risk of bias across studies | 15 | Specify any assessment of risk of bias that may affect the cumulative evidence (e.g., publication bias, selective reporting within studies). | Statistical Analysis |
| Additional analyses | 16 | Describe methods of additional analyses if done, indicating which were pre-specified. This may include, but not be limited to, the following:   - Sensitivity or subgroup analyses; - Meta-regression analyses; - *Alternative formulations of the treatment network; and* - *Use of alternative prior distributions for Bayesian analyses (if applicable).* | Statistical Analysis |
| **RESULTS†** |  |  |  |
| Study selection | 17 | Give numbers of studies screened, assessed for eligibility, and included in the review, with reasons for exclusions at each stage, ideally with a flow diagram. | Study Selection and Characteristics |
| **Presentation of network structure** | **S3** | Provide a network graph of the included studies to enable visualization of the geometry of the treatment network. | Synthesis of Results |
| **Summary of network geometry** | **S4** | Provide a brief overview of characteristics of the treatment network. This may include commentary on the abundance of trials and randomized patients for the different interventions and pairwise comparisons in the network, gaps of evidence in the treatment network, and potential biases reflected by the network structure. | Synthesis of Results |
| Study characteristics | 18 | For each study, present characteristics for which data were extracted (e.g., study size, PICOS, follow-up period) and provide the citations. | Study Selection and Characteristics |
| Risk of bias within studies | 19 | Present data on risk of bias of each study and, if available, any outcome level assessment. | Study Selection and Characteristics |
| Results of individual studies | 20 | For all outcomes considered (benefits or harms), present, for each study: 1) simple summary data for each intervention group, and 2) effect estimates and confidence intervals. *Modified approaches may be needed to deal with information from larger networks.* | Primary Outcomes,  Pooled Analysis,  Secondary Outcomes |
| Synthesis of results | 21 | Present results of each meta-analysis done, including confidence/credible intervals. *In larger networks, authors may focus on comparisons versus a particular comparator (e.g. placebo or standard care), with full findings presented in an appendix. League tables and forest plots may be considered to summarize pairwise comparisons.* If additional summary measures were explored (such as treatment rankings), these should also be presented. | Primary Outcomes,  Pooled Analysis,  Secondary Outcomes |
| **Exploration for inconsistency** | **S5** | Describe results from investigations of inconsistency. This may include such information as measures of model fit to compare consistency and inconsistency models, *P* values from statistical tests, or summary of inconsistency estimates from different parts of the treatment network. | Synthesis of Results |
| Risk of bias across studies | 22 | Present results of any assessment of risk of bias across studies for the evidence base being studied. | Primary Outcomes,  Pooled Analysis,  Secondary Outcomes |
| Results of additional analyses | 23 | Give results of additional analyses, if done (e.g., sensitivity or subgroup analyses, meta-regression analyses*, alternative network geometries studied, alternative choice of prior distributions for Bayesian analyses,* and so forth). | Secondary Outcomes |
|  |  |  |  |
| **DISCUSSION** |  |  |  |
| Summary of evidence | 24 | Summarize the main findings, including the strength of evidence for each main outcome; consider their relevance to key groups (e.g., healthcare providers, users, and policy-makers). | Discussion |
| Limitations | 25 | Discuss limitations at study and outcome level (e.g., risk of bias), and at review level (e.g., incomplete retrieval of identified research, reporting bias). *Comment on the validity of the assumptions, such as transitivity and consistency. Comment on any concerns regarding network geometry (e.g., avoidance of certain comparisons).* | Limitations |
| Conclusions | 26 | Provide a general interpretation of the results in the context of other evidence, and implications for future research. | Conclusions |
|  |  |  |  |
| **FUNDING** |  |  |  |
| Funding | 27 | Describe sources of funding for the systematic review and other support (e.g., supply of data); role of funders for the systematic review. This should also include information regarding whether funding has been received from manufacturers of treatments in the network and/or whether some of the authors are content experts with professional conflicts of interest that could affect use of treatments in the network. | Funding |

**2. Search Strategy for Databases**

We search PubMed, Embase, the Cochrane Registry of Clinical Trials, Web of Science, the ClinicalTrials.gov, China National Knowledge Infrastructure (CNKI), Wanfang, Vip, China Biomedical Literature, and Chinese Clinical Trial Registry (ChiCTR) from inception to September 24, 2024. The search strategy is as follows:

**Search strategy of PubMed database**

| **Search** | **Query** | **Items found** |
| --- | --- | --- |
| #1 | (((("Hydrocephalus"[Mesh]) OR (Hydrocephaly[Title/Abstract])) OR (Communicating Hydrocephalus[Title/Abstract])) OR (Hydrocephalus, Communicating[Title/Abstract])) OR (Idiopathic normal pressure hydrocephalus[Title/Abstract]) | 27932 |
| #2 | (((((((((((((("Therapeutics"[Mesh]) OR (Therapeutic[Title/Abstract])) OR (Therapy[Title/Abstract])) OR (Therapies[Title/Abstract])) OR (Treatment[Title/Abstract])) OR (Treatments[Title/Abstract])) OR (Surgery[Title/Abstract])) OR (ventriculoperitoneal shunt[Title/Abstract])) OR (ventriculoatrial shunt[Title/Abstract])) OR (lumboperitoneal shunt[Title/Abstract])) OR (ventriculopleural shunt[Title/Abstract])) OR (endoscopic third ventriculostomy[Title/Abstract])) OR (internal ventricular cerebrospinal fluid shunt[Title/Abstract])) OR (choroid plexus coagulation[Title/Abstract])) OR (choroid plexus cauterization[Title/Abstract]) | 11793478 |
| #3 | (clinical[Title/Abstract] AND trial[Title/Abstract]) OR clinical trials as topic[MeSH Terms] OR clinical trial[Publication Type] OR random*[Title/Abstract] OR random allocation[MeSH Terms] OR therapeutic use[MeSH Subheading] | 6610764 |
| #4 | #1 AND #2 AND #3 | 4107 |
| #5 | (((((complications[Title/Abstract]) OR (efficacy[Title/Abstract])) OR (safety[Title/Abstract])) OR (mortality[Title/Abstract])) OR (infection[Title/Abstract])) OR (revision[Title/Abstract]) | 4637830 |
| #6 | #4 AND #5 | 2029 |

**Search strategy of EMBASE database**

| **Search** | **Query** | **Items found** |
| --- | --- | --- |
| #1 | ‘communicating hydrocephalus’/exp OR ‘hydrocephalus, communicating’:ab,ti OR ‘idiopathic normal pressure hydrocephalus’:ab,ti | 4340 |
| #2 | ‘therapy’/exp OR ‘Therapeutics’:ab,ti OR ‘Therapies’:ab,ti OR ‘Treatment’:ab,ti OR ‘Treatments’:ab,ti OR ‘surgery’/exp OR ‘ventriculoperitoneal shunt’:ab,ti OR ‘ventriculoatrial shunt’:ab,ti OR ‘lumboperitoneal shunt’:ab,ti OR ‘ventriculopleural shunt’:ab,ti OR ‘endoscopic third ventriculostomy’:ab,ti OR ‘internal ventricular cerebrospinal fluid shunt’:ab,ti OR ‘choroid plexus coagulation’:ab,ti OR ‘choroid plexus cauterization’:ab,ti | 18335526 |
| #3 | random:ab,ti OR placebo:ab,ti OR double-blind:ab,ti | 913431 |
| #4 | #1 AND #2 AND #3 | 39 |

**Search strategy of Cochrane Central Register of Controlled Trials**

| **Search** | **Query** | **Items found** |
| --- | --- | --- |
| #1 | MeSH descriptor: [Hydrocephalus] explode all trees | 313 |
| #2 | (communicating hydrocephalus):ti,ab,kw OR (hydrocephalus, communicating):ti,ab,kw OR (idiopathic normal pressure hydrocephalus):ti,ab,kw OR (Hydrocephaly):ti,ab,kw | 925 |
| #3 | #1 OR #3 | 925 |
| #4 | MeSH descriptor: [Therapeutics] explode all trees | 433529 |
| #5 | MeSH descriptor: [General Surgery] explode all trees | 508 |
| #6 | (Therapeutic):ti,ab,kw OR (Therapies):ti,ab,kw (Therapeutics):ti,ab,kw OR (Therapies):ti,ab,kw OR (Treatment):ti,ab,kw OR (Treatments):ti,ab,kw OR (ventriculoperitoneal shunt):ti,ab,kw OR (ventriculoatrial shunt):ti,ab,kw OR (lumboperitoneal shunt):ti,ab,kw OR (ventriculopleural shunt):ti,ab,kw OR (endoscopic third ventriculostomy):ti,ab,kw OR (internal ventricular cerebrospinal fluid shunt):ti,ab,kw OR (choroid plexus coagulation):ti,ab,kw OR (choroid plexus cauterization):ti,ab,kw | 1126167 |
| #7 | #4 OR #5 OR #6 | 1248312 |
| #8 | #3 AND #7 | 671 |

**Search strategy of Web of Science**

| **Search** | **Query** | **Items found** |
| --- | --- | --- |
| #1 | TS=(Hydrocephaly OR Communicating Hydrocephalus OR Hydrocephalus, Communicating OR Idiopathic normal pressure hydrocephalus) | 4081 |
| #2 | TS=(Therapeutic* OR Therapy OR Therapies OR Treatment* OR Surgery OR ventriculoperitoneal shunt OR ventriculoatrial shunt OR lumboperitoneal shunt OR ventriculopleural shunt OR endoscopic third ventriculostomy OR internal ventricular cerebrospinal fluid shunt OR choroid plexus coagulation OR choroid plexus cauterization) | [10080695](https://www.webofscience.com/wos/woscc/summary/746ebb8b-2629-4130-a091-048053f99b3a-010adc8379/relevance/1) |
| #3 | TS=(randomized controlled trial OR randomized OR Placebo) | [1286423](https://www.webofscience.com/wos/woscc/summary/eefa3481-9db2-48c0-8864-9b74a67205df-010adc8c4a/relevance/1) |
| #4 | #1 AND #2 AND #3 | 69 |

**Search strategy for registered trials**

| **Clinicaltrials.gov search strategy** | |
| --- | --- |
| 1 | Communicating hydrocephalus [Condition/disease] |
| 2 | Interventional OR Observational [Study Type] |
| 8 | 1 and 2 |
| **Chictr.org.cn search strategy** | |
| 1 | Hydrocephalus [Scientific title] |

**3. Definition of Outcomes**

**3.1 Efficacy: Favorable outcome**

| **Study ID** | **Definition** |
| --- | --- |
| Huang 2017,  Zhang 2019,  Chen 2022,  Wu 2022,  Wang 2021,  Liang 2020,  Wang 2020,  Li 2017,  Guo 2019,  Du 2018,  Huang 2015,  Lu 2017,  Wu 2020,  Zang 2021,  Li 2020b,  Zhang 2018,  Hu 2018,  Cheng 2016. | Excellent: The patient's consciousness, intelligence and mobility improved significantly, and clinical symptoms such as headache, dizziness, vomiting, and papilledema nearly disappeared; Cranial CT or MRI examinations reveal a significant reduction in the size of the ventricular system, with no or minimal subdural effusion.  Good: Clinical symptoms mentioned above are partially alleviated. Cranial CT or MRI examinations indicate a slight reduction in the size of the ventricular system, accompanied by brain tissue recruitment at the site of hydrocephalus.  Poor: No relief of clinical symptoms and no changes or enlargement of the ventricular system on CT or MRI imaging.  Favorable Outcome Rate = (Number of Excellent Cases + Number of Good Cases) / Total Number of Cases × 100 % |
| Kulkarni 2017,  Goyal 2014,  Aranha 2018,  Su 2013,  Gong 2021,  Han 2016,  Ye 2017. | Favorable outcome: Clinical improvement, requiring no further surgical intervention |
| Li 2020a | Criteria for Efficacy Assessment Based on National Institute of Health stroke scale (NIHSS):  Recovery: Clinical symptoms nearly disappear, imaging shows a significant reduction in ventricular size, NIHSS score decreases by more than 90%, and disability level is 0;  Significantly Effective: Clinical symptoms significantly improve, imaging indicates a notable reduction in ventricular size, NIHSS score decreases by 46% to 90%, and disability level is between I and III;  Effective: Clinical symptoms show improvement, imaging suggests a partial reduction in ventricular size with re-expansion of brain tissue in the hydrocephalic area, and NIHSS score decreases by 18% to 45%;  Ineffective: No improvement in clinical symptoms, with an NIHSS score reduction of less than 18%;  Favorable Outcome Rate = (Number of Recovery Cases + Number of Significantly Effective Cases + Number of Effective Cases) / Total Number of Cases×100% |
| Liu 2023 | Criteria for Efficacy Assessment Based on NIHSS:  Excellent: symptoms of the disease basically disappeared, imaging results showed that the patient's ventricular size returned to normal, no ventricular oedema was seen, and the NIHSS score was reduced by ≥46% compared with the preoperative period;  Good: significant reduction of ventricular oedema, reduction of ventricles, NIHSS score 18%-45% lower than preoperative;  Poor: Fails to meet the criteria for "Excellent" or "Good."  Favorable Outcome Rate = (Number of Excellent Cases + Number of Good Cases) / Total Number of Cases × 100 % |
| Li 2023 | Effective: Patients with clinical relief with or without reduction of the ventricular system on imaging;  Ineffective: Patients with no clinical relief with or without enlargement of the ventricular system on imaging.  Favorable Outcome Rate = Number of Effective Cases / Total Number of Cases ×  100 % |
| Chen 2023 | Cure: Complete disappearance of clinical symptoms such as dizziness and vomiting, and normalization of the lesion area on imaging;  Significantly effective: Clinical symptoms significantly improved; ventricles significantly reduced;  Effective: Clinical symptoms improved; ventricular reduction is not obvious;  Ineffective: No improvement in clinical symptoms and no significant difference between the ventricles and those before surgery.  Favorable Outcome Rate = (Number of Cure Cases + Number of Significantly  effective Cases + Number of Effective Cases) / Total Number of Cases×100% |
| Raut 2024 | Favorable outcome: Restored to Vellore Grading I |
| Pinto 2013 | Favorable outcome: A patient have at least a 2 points higher score on the NPH Scale. |

**3.2 Safety: Complications**

The complications will cover at least two adverse events including: infection, seizure, under-drainage, over-drainage, shunt malfunction/obstruction, intracranial haemorrhage, subdural haemorrhage/fluid collection, radicular pain, incisional hernia, abdominal adhesions, cerebral vasospasm, and death.

**4. Details of the Bayesian model specifications**

Bayesian NMAs were conducted using the GeMTC (version 1.0.2) package in R (version 4.3.2) with Markov Chain Monte Carlo (MCMC) method. Uninformative prior distributions were applied for the treatment effects, with a minimally informative prior used for the common standard deviation parameter. Four MCMC chains were set for the initial value, with each chain undergoing 50,000 iterations. To exclude initial value bias, we discarded the first 10,000 annealings and commenced sampling from iteration 10,001. A thinning interval of 10 was applied to reduce autocorrelation, retaining every 10th sample from each MCMC chain. Trace, density and Brooks-Gelman-Rubin diagnosis plots were utilized to visually examine convergence. Model fit was evaluated through the posterior total residual deviance and unconstrained data points. Both random-effects (RE) and fixed-effects (FE) models were used to pool network results. Model selection generally be guided by both statistical and clinical heterogeneity. When between-study results are consistent and clinical heterogeneity is minimal, a fixed-effects model is often considered more appropriate. Within our Bayesian framework, model choice was primarily informed by the Deviance Information Criterion (DIC), where lower values indicate better model fit. When the DIC difference between models was less than 5, suggesting comparable fit, we preferred the fixed-effects model.

| **MCMC Characteristic** | **Value** |
| --- | --- |
| Chains | 4 |
| Burn-in iterations | 10000 |
| Sample iterations | 50000 |
| Thinning factor | 10 |

**eTable 1. Risk of Bias Assessment**

**Panel A**

**Version 2 of the Cochrane tool for assessing risk of bias in randomised trial (RoB2)**

| **Study ID** | **Randomisation process** | **Deviations from  the intended interventions** | **Missing outcome data** | **Measurement of  the outcome** | **Selection of  the reported result** | **Overall risk of bias ^a^** |
| --- | --- | --- | --- | --- | --- | --- |
| Aranha 2018 | **Low risk** | **Low risk** | **Some concerns** | **Low risk** | **Some concerns** | **Some concerns** |
| Chen 2022 | **Low risk** | **Low risk** | **Low risk** | **Low risk** | **Low risk** | **Low risk** |
| Chen 2023 | **Low risk** | **Low risk** | **Low risk** | **Low risk** | **Low risk** | **Low risk** |
| Cheng 2016 | **Some concerns** | **Low risk** | **Low risk** | **Low risk** | **Some concerns** | **Some concerns** |
| Du 2018 | **Low risk** | **Low risk** | **Low risk** | **Low risk** | **Low risk** | **Low risk** |
| Gong 2021 | **Low risk** | **Low risk** | **Low risk** | **Low risk** | **Low risk** | **Low risk** |
| Goyal 2014 | **Some concerns** | **Low risk** | **Some concerns** | **Low risk** | **Low risk** | **Some concerns** |
| Guo 2019 | **Low risk** | **Low risk** | **Low risk** | **Low risk** | **Low risk** | **Low risk** |
| Han 2016 | **Low risk** | **Low risk** | **Low risk** | **Low risk** | **Low risk** | **Low risk** |
| Hu 2018 | **Low risk** | **Low risk** | **Low risk** | **Low risk** | **Low risk** | **Low risk** |
| Huang 2015 | **Some concerns** | **Low risk** | **Low risk** | **Some concerns** | **Low risk** | **Some concerns** |
| Huang 2017 | **Low risk** | **Low risk** | **Low risk** | **Low risk** | **Low risk** | **Low risk** |
| Kulkarni 2017 | **Low risk** | **Low risk** | **Low risk** | **Low risk** | **Low risk** | **Low risk** |
| Li 2017 | **Low risk** | **Low risk** | **Low risk** | **Low risk** | **Low risk** | **Low risk** |
| Li 2020a | **Low risk** | **Low risk** | **Low risk** | **Low risk** | **Low risk** | **Low risk** |
| Li 2020b | **Low risk** | **Low risk** | **Low risk** | **Low risk** | **Low risk** | **Low risk** |
| Li 2023 | **Low risk** | **Low risk** | **Low risk** | **Low risk** | **Low risk** | **Low risk** |
| Liang 2020 | **Low risk** | **Low risk** | **Low risk** | **Low risk** | **Low risk** | **Low risk** |
| Liu 2019 | **Some concerns** | **Low risk** | **High risk** | **Some concerns** | **Some concerns** | **High risk** |
| Liu 2023 | **Some concerns** | **Low risk** | **Some concerns** | **Low risk** | **Low risk** | **Some concerns** |
| Lu 2017 | **Low risk** | **Low risk** | **Low risk** | **Low risk** | **Low risk** | **Low risk** |
| Pinto 2013 | **Low risk** | **Low risk** | **Low risk** | **Low risk** | **Low risk** | **Low risk** |
| Punchak 2019 | **Low risk** | **Low risk** | **Low risk** | **Low risk** | **Low risk** | **Low risk** |
| Raut 2024 | **Low risk** | **Low risk** | **Low risk** | **Low risk** | **Low risk** | **Low risk** |
| Su 2013 | **Low risk** | **Low risk** | **Low risk** | **Low risk** | **Low risk** | **Low risk** |
| Wang 2020 | **Some concerns** | **Low risk** | **Some concerns** | **Low risk** | **Low risk** | **Some concerns** |
| Wang 2021 | **Low risk** | **Low risk** | **Low risk** | **Low risk** | **Low risk** | **Low risk** |
| Wu 2020 | **Low risk** | **Low risk** | **Low risk** | **Low risk** | **Low risk** | **Low risk** |
| Wu 2022 | **Low risk** | **Low risk** | **Low risk** | **Low risk** | **Low risk** | **Low risk** |
| Xiong 2014 | **Some concerns** | **Low risk** | **Low risk** | **Low risk** | **Some concerns** | **Some concerns** |
| Ye 2017 | **Some concerns** | **Low risk** | **Some concerns** | **Low risk** | **Low risk** | **Some concerns** |
| Zang 2021 | **Low risk** | **Low risk** | **Low risk** | **Low risk** | **Low risk** | **Low risk** |
| Zhang 2018 | **Low risk** | **Low risk** | **Low risk** | **Low risk** | **Low risk** | **Low risk** |
| Zhang 2019 | **Low risk** | **Low risk** | **Low risk** | **Low risk** | **Low risk** | **Low risk** |

^a^ The overall risk of bias judgement was classified as follows: Low risk of bias: the study is judged to be at low risk of bias for all domains for this result; Some concerns: the study is judged to raise some concerns in at least one domain for this result, but not to be at high risk of bias for any domain; High risk of bias: the study is judged to be at high risk of bias in at least one domain for this result, or the study is judged to have some concerns for multiple domains in a way that substantially lowers confidence in the result.

**Panel B**

**Risk of bias contribution by intervention group**

Abbreviations: VPS, ventriculoperitoneal; LPS, lumboperitoneal shunt; LPS+LS, lumboperitoneal shunt with laparoscope; ETV, endoscopic third ventriculostomy; ETV+CPC, endoscopic third ventriculostomy with choroid plexus cauterization.

**Panel C**

**Overall risk of bias by treatment comparison**

Abbreviations: VPS, ventriculoperitoneal; LPS, lumboperitoneal shunt; LPS+LS, lumboperitoneal shunt with laparoscope; ETV, endoscopic third ventriculostomy; ETV+CPC, endoscopic third ventriculostomy with choroid plexus cauterization.

**eTable 2. CINeMA Assessment**

1. **Favorable outcome**

| **Comparison** | **Number of studies** | **Within-study bias** | **Reporting bias** | **Indirectness** | **Imprecision** | **Heterogeneity** | **Incoherence** | **Confidence rating** |
| --- | --- | --- | --- | --- | --- | --- | --- | --- |
| **Mixed evidence** | | | | | | | | |
| **ETV vs VPS** | 8 | **No concerns** | **Low risk** | **No concerns** | **No concerns** | **No concerns** | **No concerns** | **High** |
| **ETV+CPC vs VPS** | 1 | **No concerns** | **Low risk** | **Some concerns** | **Some concerns** | **No concerns** | **No concerns** | **Moderate** |
| **LPS+LS vs LPS** | 3 | **No concerns** | **Low risk** | **No concerns** | **No concerns** | **No concerns** | **No concerns** | **High** |
| **LPS vs VPS** | 17 | **No concerns** | **Low risk** | **No concerns** | **No concerns** | **No concerns** | **No concerns** | **High** |
| **LPS+LS vs VPS** | 4 | **No concerns** | **Low risk** | **No concerns** | **No concerns** | **No concerns** | **No concerns** | **High** |
| **Indirect evidence** | | | | | | | | |
| **ETV vs ETV+CPC** | 0 | **No concerns** | **Low risk** | **Some concerns** | **Some concerns** | **No concerns** | **No concerns** | **Low** |
| **ETV vs LPS** | 0 | **No concerns** | **Low risk** | **No concerns** | **No concerns** | **Some concerns** | **No concerns** | **Moderate** |
| **ETV vs LPS+LS** | 0 | **No concerns** | **Low risk** | **No concerns** | **No concerns** | **No concerns** | **No concerns** | **High** |
| **ETV+CPC vs LPS** | 0 | **No concerns** | **Low risk** | **No concerns** | **No concerns** | **Some concerns** | **No concerns** | **Moderate** |
| **ETV+CPC vs LPS+LS** | 0 | **No concerns** | **Low risk** | **No concerns** | **No concerns** | **No concerns** | **No concerns** | **High** |

1. **Complications**

| **Comparison** | **Number of studies** | **Within-study bias** | **Reporting bias** | **Indirectness** | **Imprecision** | **Heterogeneity** | **Incoherence** | **Confidence rating** |
| --- | --- | --- | --- | --- | --- | --- | --- | --- |
| **Mixed evidence** | | | | | | | | |
| **ETV vs VPS** | 3 | **No concerns** | **Low risk** | **No concerns** | **No concerns** | **No concerns** | **No concerns** | **High** |
| **ETV+CPC vs VPS** | 1 | **No concerns** | **Low risk** | **Some concerns** | **Major concerns** | **No concerns** | **Some concerns** | **Low** |
| **LPS+LS vs LPS** | 2 | **No concerns** | **Low risk** | **No concerns** | **Some concerns** | **No concerns** | **Some concerns** | **Moderate** |
| **LPS vs VPS** | 15 | **No concerns** | **Low risk** | **No concerns** | **No concerns** | **No concerns** | **No concerns** | **High** |
| **LPS+LS vs VPS** | 4 | **No concerns** | **Low risk** | **No concerns** | **No concerns** | **No concerns** | **No concerns** | **High** |
| **Indirect evidence** | | | | | | | | |
| **ETV vs ETV+CPC** | 0 | **No concerns** | **Low risk** | **Some concerns** | **No concerns** | **No concerns** | **Major concerns** | **Low** |
| **ETV vs LPS** | 0 | **No concerns** | **Low risk** | **No concerns** | **Major concerns** | **No concerns** | **Major concerns** | **Low** |
| **ETV vs LPS+LS** | 0 | **No concerns** | **Low risk** | **No concerns** | **Major concerns** | **No concerns** | **Major concerns** | **Low** |
| **ETV+CPC vs LPS** | 0 | **No concerns** | **Low risk** | **No concerns** | **No concerns** | **No concerns** | **Major concerns** | **Low** |
| **ETV+CPC vs LPS+LS** | 0 | **No concerns** | **Low risk** | **No concerns** | **No concerns** | **Some concerns** | **Major concerns** | **Low** |

1. **Infection**

| **Comparison** | **Number of studies** | **Within-study bias** | **Reporting bias** | **Indirectness** | **Imprecision** | **Heterogeneity** | **Incoherence** | **Confidence rating** |
| --- | --- | --- | --- | --- | --- | --- | --- | --- |
| **Mixed evidence** | | | | | | | | |
| **ETV vs VPS** | 5 | **No concerns** | **Low risk** | **Some concerns** | **No concerns** | **No concerns** | **No concerns** | **Moderate** |
| **ETV+CPC vs VPS** | 1 | **No concerns** | **Low risk** | **Some concerns** | **Major concerns** | **No concerns** | **No concerns** | **Low** |
| **LPS+LS vs LPS** | 3 | **No concerns** | **Low risk** | **No concerns** | **Some concerns** | **No concerns** | **No concerns** | **Moderate** |
| **LPS vs VPS** | 16 | **No concerns** | **Low risk** | **No concerns** | **No concerns** | **No concerns** | **No concerns** | **High** |
| **LPS+LS vs VPS** | 4 | **No concerns** | **Low risk** | **No concerns** | **No concerns** | **No concerns** | **No concerns** | **High** |
| **Indirect evidence** | | | | | | | | |
| **ETV vs ETV+CPC** | 0 | **No concerns** | **Low risk** | **Some concerns** | **Major concerns** | **No concerns** | **No concerns** | **Low** |
| **ETV vs LPS** | 0 | **No concerns** | **Low risk** | **No concerns** | **Major concerns** | **No concerns** | **No concerns** | **Moderate** |
| **ETV vs LPS+LS** | 0 | **No concerns** | **Low risk** | **No concerns** | **Major concerns** | **No concerns** | **No concerns** | **Moderate** |
| **ETV+CPC vs LPS** | 0 | **No concerns** | **Low risk** | **No concerns** | **Major concerns** | **No concerns** | **No concerns** | **Moderate** |
| **ETV+CPC vs LPS+LS** | 0 | **No concerns** | **Low risk** | **No concerns** | **Major concerns** | **No concerns** | **No concerns** | **Moderate** |

1. **Revision**

| **Comparison** | **Number of studies** | **Within-study bias** | **Reporting bias** | **Indirectness** | **Imprecision** | **Heterogeneity** | **Incoherence** | **Confidence rating** |
| --- | --- | --- | --- | --- | --- | --- | --- | --- |
| **Mixed evidence** | | | | | | | | |
| **ETV vs VPS** | 2 | **Some concerns** | **Low risk** | **Some concerns** | **Major concerns** | **No concerns** | **No concerns** | **Low** |
| **ETV+CPC vs VPS** | 1 | **No concerns** | **Low risk** | **Some concerns** | **Major concerns** | **No concerns** | **No concerns** | **Low** |
| **LPS+LS vs LPS** | 1 | **No concerns** | **Low risk** | **No concerns** | **Major concerns** | **No concerns** | **No concerns** | **Moderate** |
| **LPS vs VPS** | 1 | **No concerns** | **Low risk** | **No concerns** | **No concerns** | **Major concerns** | **No concerns** | **Moderate** |
| **LPS+LS vs VPS** | 2 | **No concerns** | **Low risk** | **No concerns** | **No concerns** | **Some concerns** | **No concerns** | **Moderate** |
| **Indirect evidence** | | | | | | | | |
| **ETV vs ETV+CPC** | 0 | **No concerns** | **Low risk** | **Some concerns** | **Major concerns** | **No concerns** | **No concerns** | **Low** |
| **ETV vs LPS** | 0 | **No concerns** | **Low risk** | **No concerns** | **No concerns** | **Major concerns** | **No concerns** | **Moderate** |
| **ETV vs LPS+LS** | 0 | **No concerns** | **Low risk** | **No concerns** | **No concerns** | **Major concerns** | **No concerns** | **Moderate** |
| **ETV+CPC vs LPS** | 0 | **No concerns** | **Low risk** | **No concerns** | **No concerns** | **Major concerns** | **No concerns** | **Moderate** |
| **ETV+CPC vs LPS+LS** | 0 | **No concerns** | **Low risk** | **No concerns** | **No concerns** | **Major concerns** | **No concerns** | **Moderate** |

1. **Seizures**

| **Comparison** | **Number of studies** | **Within-study bias** | **Reporting bias** | **Indirectness** | **Imprecision** | **Heterogeneity** | **Incoherence** | **Confidence rating** |
| --- | --- | --- | --- | --- | --- | --- | --- | --- |
| **Mixed evidence** | | | | | | | | |
| **ETV+CPC vs VPS** | 1 | **Low risk** | **Low risk** | **Some concerns** | **Major concerns** | **No concerns** | **No concerns** | **Low** |
| **LPS+LS vs LPS** | 1 | **No concerns** | **Low risk** | **No concerns** | **Major concerns** | **No concerns** | **No concerns** | **Moderate** |
| **LPS vs VPS** | 7 | **No concerns** | **Low risk** | **No concerns** | **Some concerns** | **No concerns** | **No concerns** | **Moderate** |
| **LPS+LS vs VPS** | 2 | **No concerns** | **Low risk** | **No concerns** | **Major concerns** | **No concerns** | **Major concerns** | **Low** |
| **Indirect evidence** | | | | | | | | |
| **ETV+CPC vs LPS** | 0 | **Low risk** | **Low risk** | **No concerns** | **Major concerns** | **No concerns** | **No concerns** | **Moderate** |
| **ETV+CPC vs LPS+LS** | 0 | **No concerns** | **Low risk** | **No concerns** | **Major concerns** | **No concerns** | **No concerns** | **Moderate** |

1. **Operation time**

| **Comparison** | **Number of studies** | **Within-study bias** | **Reporting bias** | **Indirectness** | **Imprecision** | **Heterogeneity** | **Incoherence** | **Confidence rating** |
| --- | --- | --- | --- | --- | --- | --- | --- | --- |
| **Mixed evidence** | | | | | | | | |
| **ETV vs VPS** | 2 | **No concerns** | **Low risk** | **Some concerns** | **No concerns** | **No concerns** | **Some concerns** | **Moderate** |
| **LPS+LS vs LPS** | 1 | **No concerns** | **Low risk** | **No concerns** | **No concerns** | **Some concerns** | **Some concerns** | **Moderate** |
| **LPS vs VPS** | 6 | **Some concerns** | **Low risk** | **No concerns** | **No concerns** | **No concerns** | **Some concerns** | **Moderate** |
| **Indirect evidence** | | | | | | | | |
| **ETV vs LPS** | 0 | **No concerns** | **Low risk** | **No concerns** | **No concerns** | **Some concerns** | **Major concerns** | **Low** |
| **ETV vs LPS+LS** | 0 | **No concerns** | **Low risk** | **No concerns** | **Major concerns** | **No concerns** | **Major concerns** | **Low** |
| **LPS+LS vs VPS** | 0 | **No concerns** | **Low risk** | **No concerns** | **No concerns** | **No concerns** | **Major concerns** | **Moderate** |

1. **Duration of hospitalisation**

| **Comparison** | **Number of studies** | **Within-study bias** | **Reporting bias** | **Indirectness** | **Imprecision** | **Heterogeneity** | **Incoherence** | **Confidence rating** |
| --- | --- | --- | --- | --- | --- | --- | --- | --- |
| **Mixed evidence** | | | | | | | | |
| **ETV vs VPS** | 2 | **No concerns** | **Low risk** | **No concerns** | **No concerns** | **No concerns** | **Major concerns** | **Moderate** |
| **LPS vs VPS** | 4 | **Some concerns** | **Low risk** | **No concerns** | **No concerns** | **No concerns** | **Some concerns** | **Moderate** |
| **Indirect evidence** | | | | | | | | |
| **ETV vs LPS** | 0 | **No concerns** | **Low risk** | **No concerns** | **No concerns** | **No concerns** | **Major concerns** | **Moderate** |

**eTable 3. Assessment of Network Model Fit**

| **Analysis** | **FE consistency model** | | | | **RE consistency model** | | | | **FE inconsistency model** | | | | **RE inconsistency model** | | | | **Data points** |
| --- | --- | --- | --- | --- | --- | --- | --- | --- | --- | --- | --- | --- | --- | --- | --- | --- | --- |
|  | **Dbar** | **pD** | **DIC** | **I^2^** | **Dbar** | **pD** | **DIC** | **I^2^** | **Dbar** | **pD** | **DIC** | **I^2^** | **Dbar** | **pD** | **DIC** | **I^2^** |  |
| **Favorable outcome** | **82.44** | **28.63** | **111.07** | **25%** | 72.52 | 35.19 | 107.71 | 15% | 66.29 | 51.70 | 117.99 | 6% | 63.32 | 51.74 | 118.06 | 7% | 63 |
| **Complications** | **54.00** | **27.16** | **81.17** | **15%** | 47.44 | 32.87 | 80.31 | 3% | 49.07 | 48.56 | 97.63 | 6% | 49.12 | 48.62 | 97.75 | 6% | 47 |
| **Infection** | **49.32** | **32.66** | **81.98** | **0%** | 49.31 | 34.43 | 83.75 | 0% | 50.11 | 49.40 | 99.51 | 0% | 49.96 | 49.26 | 99.22 | 0% | 55 |
| **Revision** | **18.40** | **10.13** | **28.53** | **29%** | 12.59 | 11.55 | 24.14 | 0% | 12.53 | 12.39 | 24.92 | 0% | 12.48 | 12.35 | 24.83 | 0% | 14 |
| **Seizures** | **20.92** | **12.85** | **33.77** | **14%** | 19.53 | 14.20 | 33.74 | 8% | 15.97 | 15.55 | 31.52 | 0% | 15.89 | 15.48 | 31.37 | 0% | 19 |
| **Operation time** | 97.28 | 13.91 | 111.18 | 83% | **17.96** | **17.59** | **35.55** | **5%** | 18.00 | 18.00 | 36.01 | 6% | 18.03 | 18.03 | 35.05 | 6% | 18 |
| **Duration of hospitalisation** | 93.25 | 10.55 | 103.80 | 84% | **15.98** | **15.50** | **31.47** | **6%** | 16.01 | 16.01 | 32.03 | 6% | 16.00 | 16.00 | 32.00 | **6%** | 16 |

Abbreviations: FE, fixed-effects; RE, Random-effects; iNPH, idiopathic normal pressure hydrocephalus; pD, sum of leverage, also known as the effective number of parameters, with higher values indicating higher complexity and less parsimony; Dbar, mean sum of residual deviance; DIC, deviance information criterion at residual (Dbar + pD). A smaller DIC value indicates better model fit. When the DIC difference between models is less than 5, we prioritized the results from the fixed-effects model. Blue, model selected for final results

**^a^** Sensitivity analyses for primary outcomes.;

**eTable 4. GRADE Assessment**

| **Certainty assessment** | | | | | | | **№ of patients** | | **Effect** | | **Certainty** |
| --- | --- | --- | --- | --- | --- | --- | --- | --- | --- | --- | --- |
| **No of studies** | **Study design** | **Risk of bias** | **Inconsistency** | **Indirectness** | **Imprecision** | **Publication bias** | **Lumbar approach** | **Cranial approach** | **Relative (95% CI)** | **Absolute (95% CI)** |  |
| **Favorable outcome** | | | | | | | | | | | |
| 20 | randomized trials | serious ^a^ | not serious | not serious | not serious | Undetected | 770/820 (93.9%) | 636/826 (77.0%) | **RR 1.23** (1.19 to 1.28) | **177 more per 1,000** (from 146 more to 216 more) | ⨁⨁⨁O  Moderate |
| **Complications** | | | | | | | | | | | |
| 18 | randomized trials | serious ^a^ | not serious | not serious | not serious | Undetected | 68/659 (10.3%) | 207/665 (31.1%) | **RR 0.33** (0.26 to 0.43) | **209 fewer per 1,000** (from 230 fewer to 177 fewer) | ⨁⨁⨁O  Moderate |
| **Infection** | | | | | | | | | | | |
| 18 | randomized trials | serious ^a^ | not serious | not serious | not serious | Undetected | 15/688 (2.2%) | 62/694 (8.9%) | **RR 0.28** (0.17 to 0.46) | **64 fewer per 1,000** (from 74 fewer to 48 fewer) | ⨁⨁⨁O  Moderate |
| **Revision** | | | | | | | | | | | |
| 3 | randomized trials | serious ^a^ | not serious | not serious | not serious | Undetected | 14/257 (5.4%) | 52/263 (19.8%) | **RR 0.28** (0.16 to 0.49) | **142 fewer per 1,000** (from 166 fewer to 101 fewer) | ⨁⨁⨁O  Moderate |
| **Seizures** | | | | | | | | | | | |
| 8 | randomized trials | serious ^a^ | not serious | not serious | not serious | Undected | 5/313 (1.6%) | 16/319 (5.0%) | **RR 0.41** (0.18 to 0.94) | **30 fewer per 1,000** (from 41 fewer to 3 fewer) | ⨁⨁⨁O Moderate |
| **Operation time** | | | | | | | | | | | |
| 4 | randomized trials | serious ^a^ | serious ^b^ | not serious | not serious | Undected | 150 | 150 | NA | MD **23.07 minutes fewer** (25.42 fewer to 20.73 fewer) | ⨁⨁OO Low |
| **Duration of hospitalization** | | | | | | | | | | | |
| 4 | randomized trials | serious ^a^ | not serious | not serious | not serious | Undected | 128 | 128 | NA | MD **8.37 days fewer** (9.4 fewer to 8.06 fewer) | ⨁⨁⨁O Moderate |

^a^ Rated down 1 level due to the lack of blinding.

^b^ Rated down 1 level due to moderate heterogeneity.

**eFigure 1. Network Plot for Secondary Outcomes**

**eFigure 2. Funnel Plots**

**Panel A**

**Comparison-Adjusted Funnel Plots**

1. **Favorable outcome**

1. **Complications**

1. **Infection**

**Panel B**

**Funnel Plots**

1. **Favorable outcome (Lumbar approach vs Cranial approach)**

**
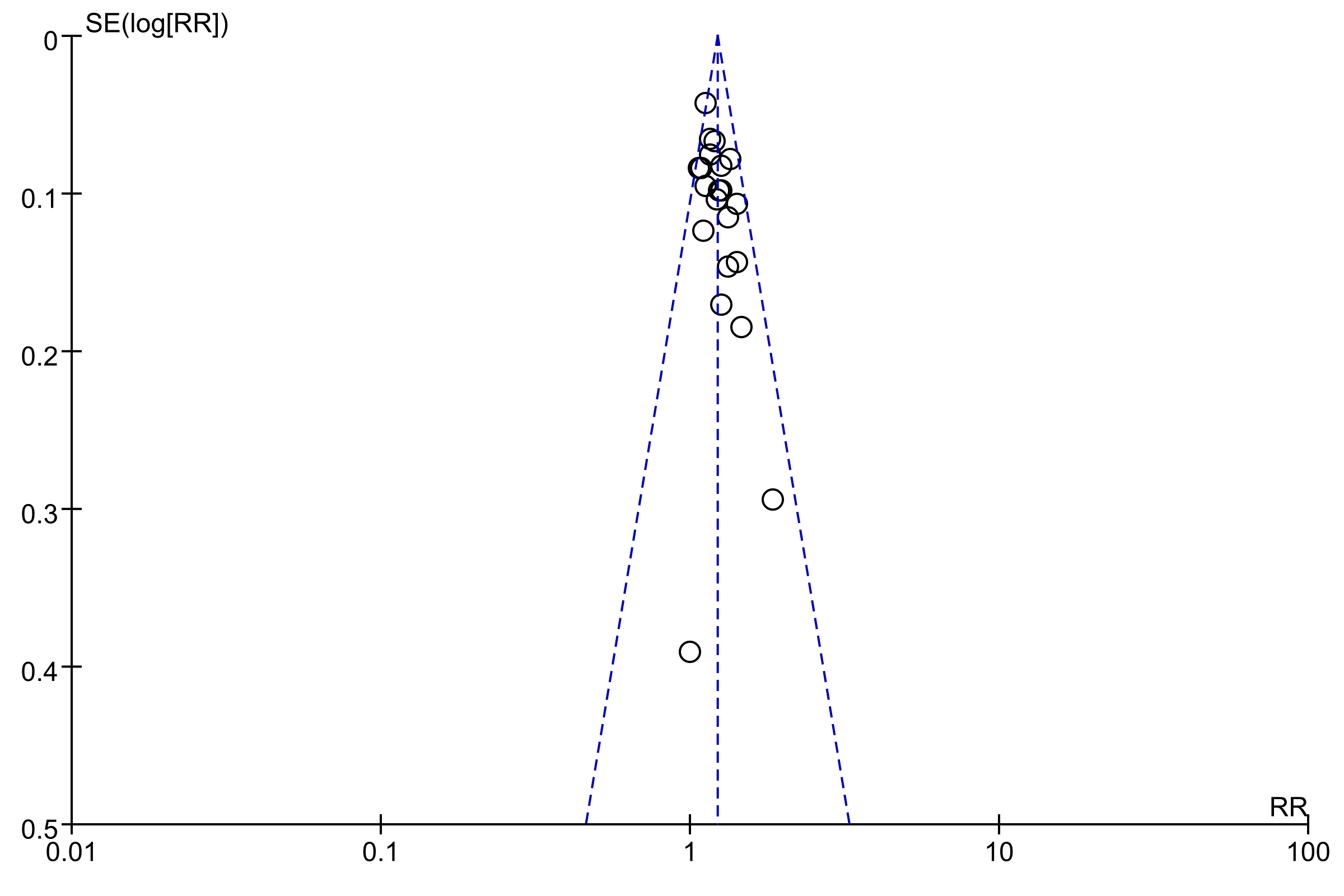
**

1. **Complications (Lumbar approach vs Cranial approach)**


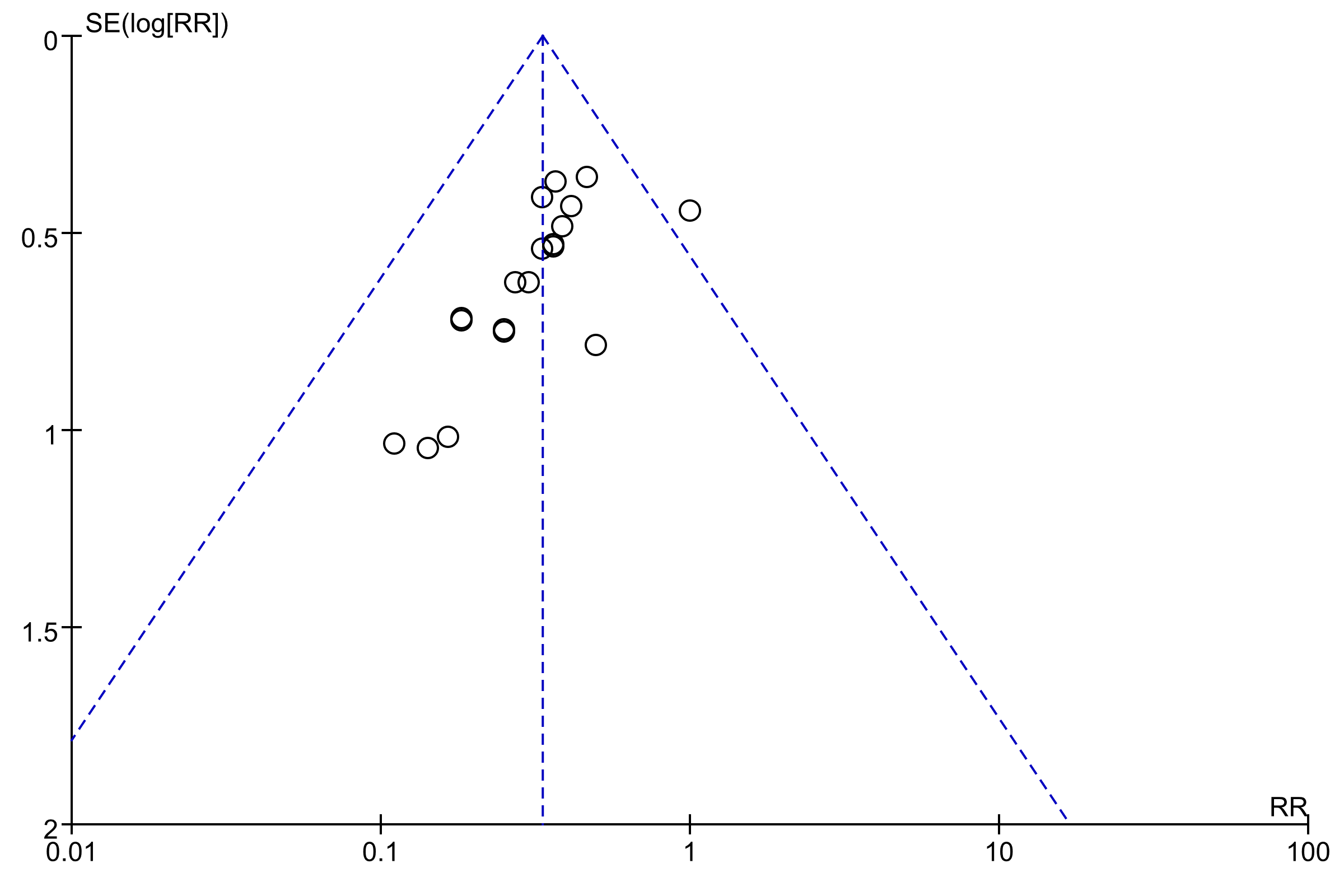


1. **Infection (Lumbar approach vs Cranial approach)**


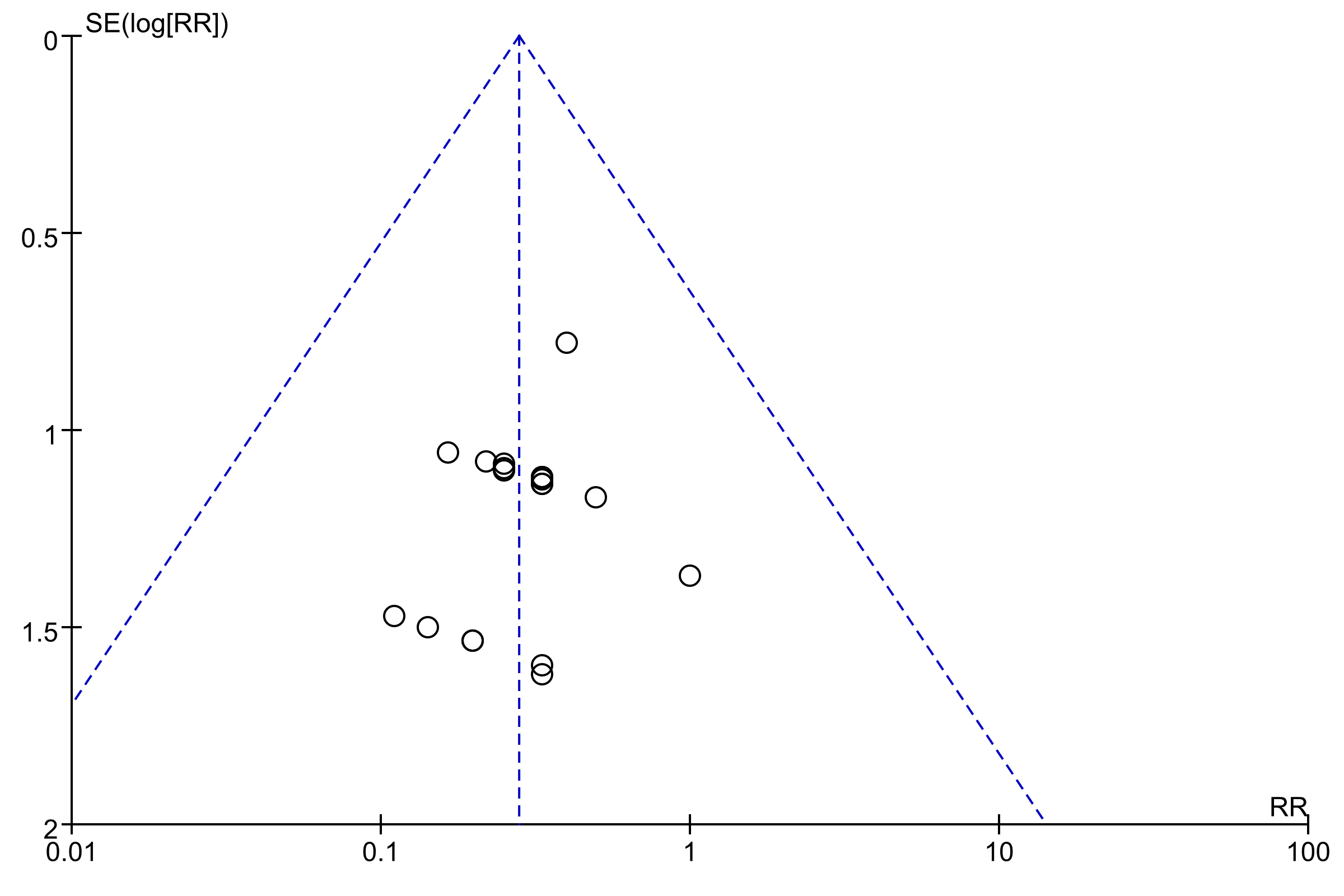


**eFigure 3. Leverage Plot of Consistency Models for Network Meta-Analysis by Outcomes**

**A. Favorable outcome (fixed-effects)**

**B. Complications (fixed-effects)**

**C. Infection (fixed-effects)**

**D. Revision (fixed-effects)**

**E. Seizures (fixed-effects)**

**F. Operation time (Random-effects)**

**G. Length of hospitalization (Random-effects)**

**eFigure 4. Inconsistency Analysis Using the Node-Splitting Approach**

1. **Favorable outcome**

1. **Complications**

1. **Infection**

1. **Revision**

1. **Seizures**

**eFigure 5. SUCRA Ranking for Primary and Secondary Outcomes**

1. **Efficacy (favorable outcome)**

**
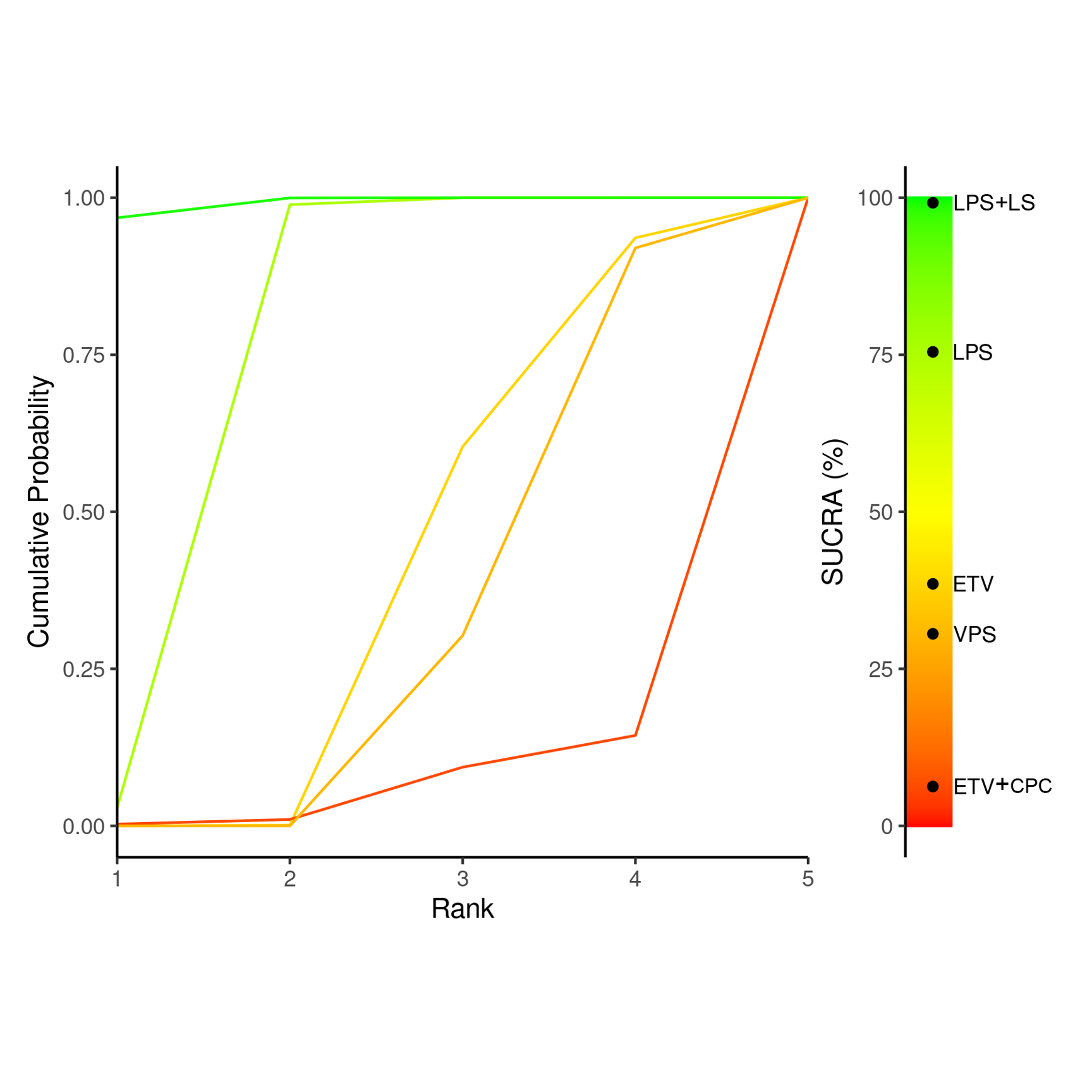
**

| **Surgical interventions** | **SUCRA score** ^a^ |
| --- | --- |
| **LPS+LS** | **99.19%** |
| LPS | 75.48% |
| ETV | 38.67% |
| VPS | 30.52% |
| **ETV+CPC** | **6.15%** |

^a^ Higher SUCRA values indicated better intervention effects.

1. **Safety (complications)**

**
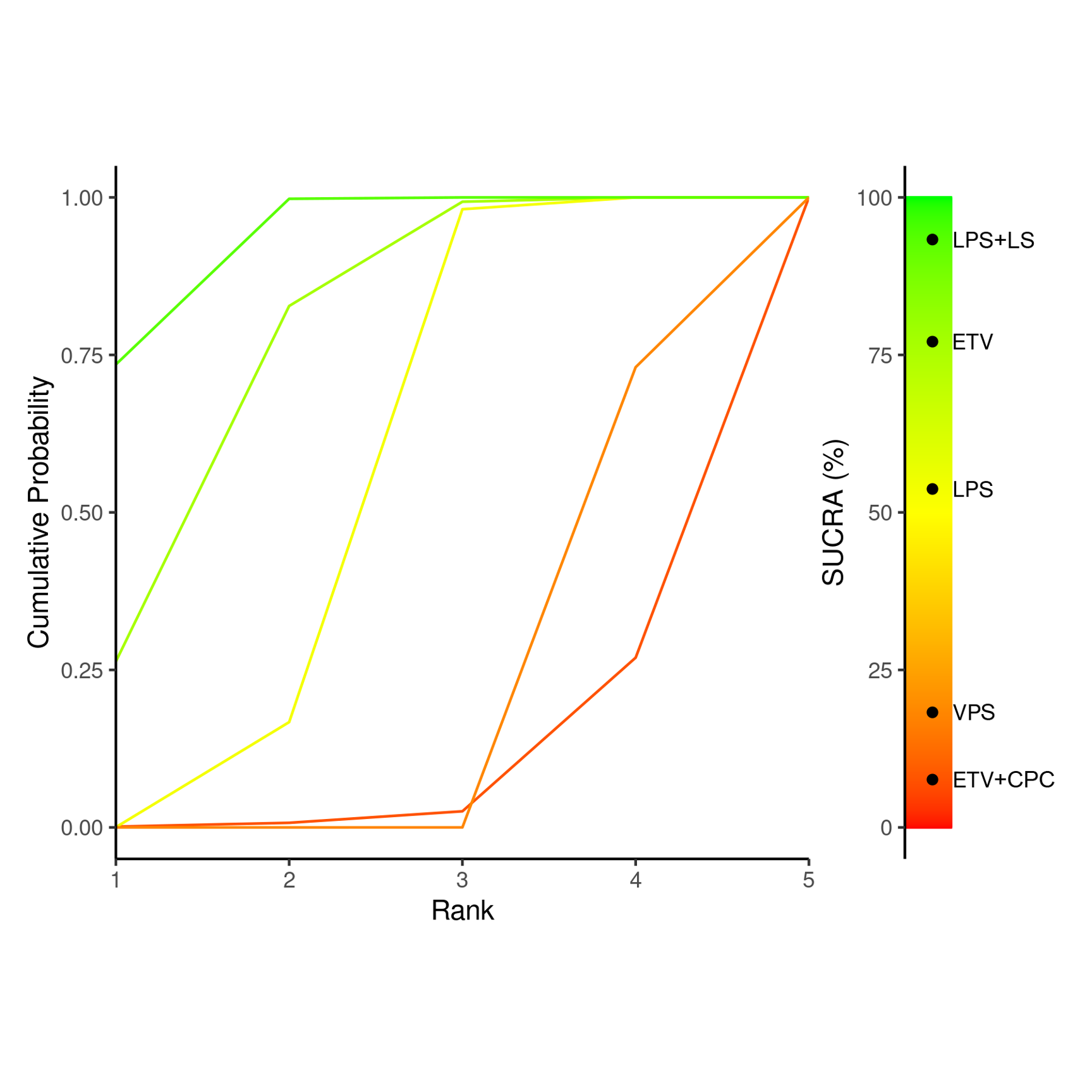
**

| **Surgical interventions** | **SUCRA score ^a^** |
| --- | --- |
| **LPS+LS** | **93.32%** |
| ETV | 77.11% |
| LPS | 53.72% |
| VPS | 18.26% |
| **ETV+CPC** | **7.59%** |

^a^ Higher SUCRA values indicated better intervention effects.

1. **Infection**


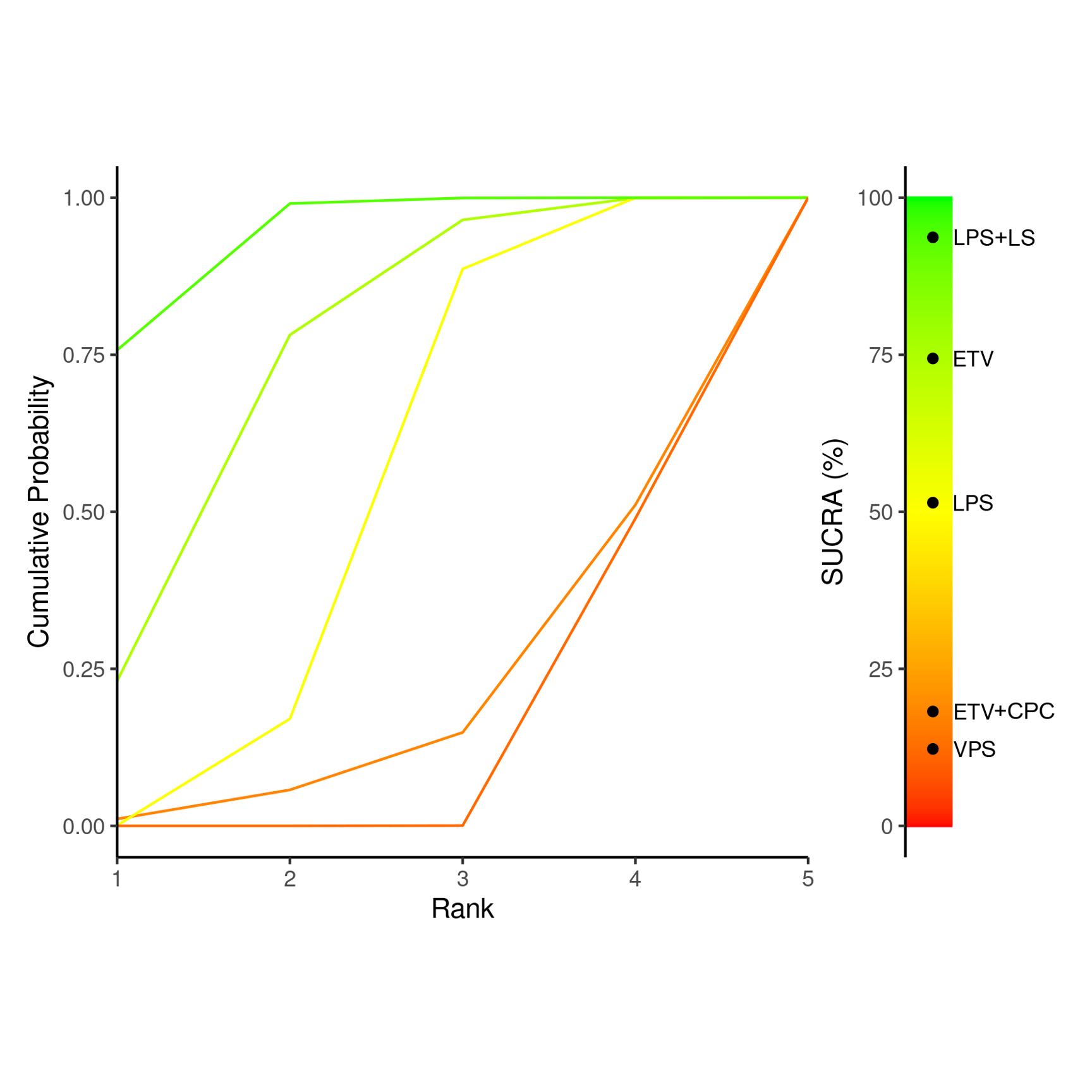


| **Surgical interventions** | **SUCRA score ^a^** |
| --- | --- |
| **LPS+LS** | **93.90%** |
| ETV | 74.24% |
| LPS | 51.30% |
| ETV+CPC | 18.33% |
| **VPS** | **12.23%** |

^a^ Higher SUCRA values indicated better intervention effects.

**D. Revision**


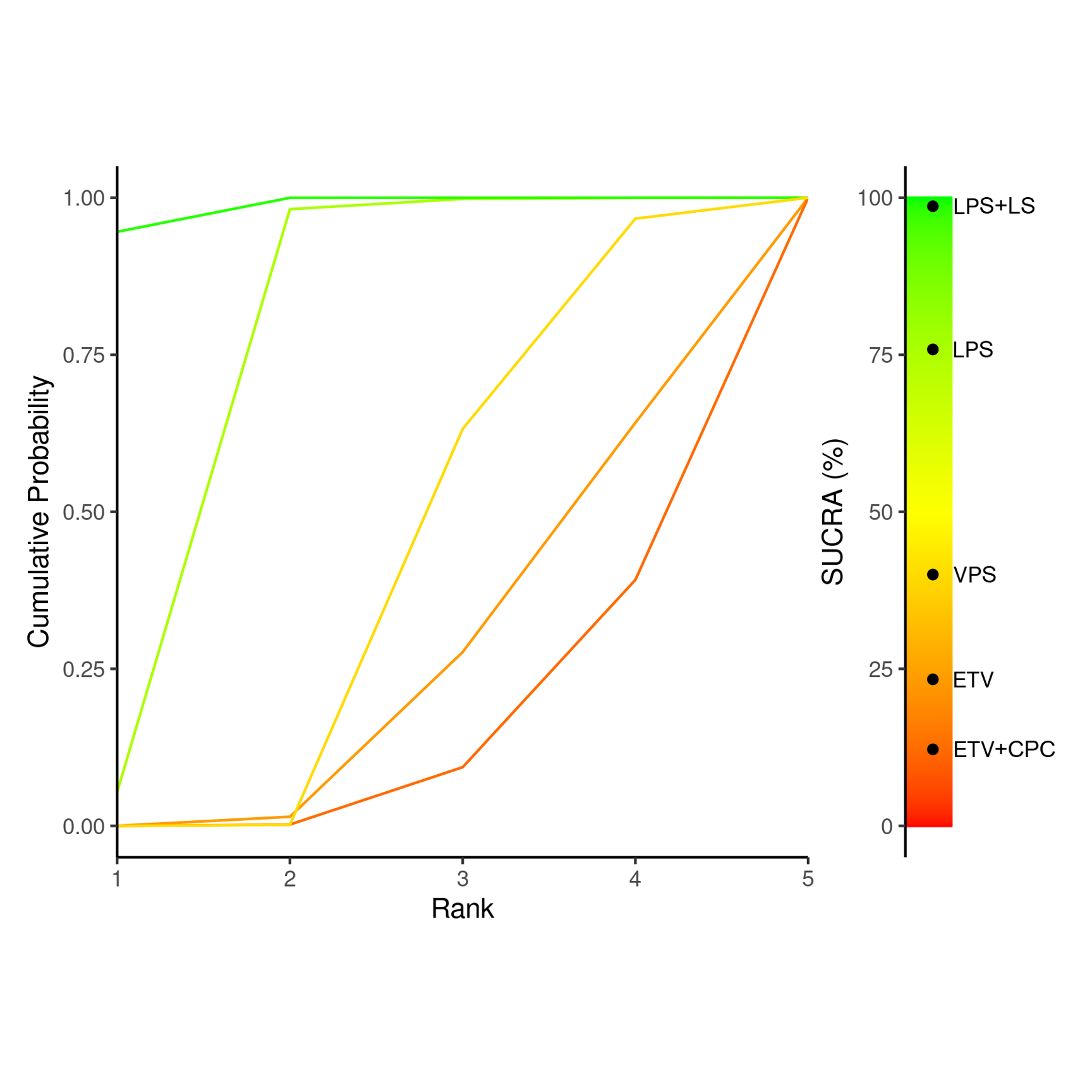


| **Surgical interventions** | **SUCRA score ^a^** |
| --- | --- |
| **LPS+LS** | **98.61%** |
| LPS | 75.89% |
| VPS | 40.06% |
| ETV | 23.17% |
| **ETV+CPC** | **12.27%** |

^a^ Higher SUCRA values indicated better intervention effects.

**E. Seizures**


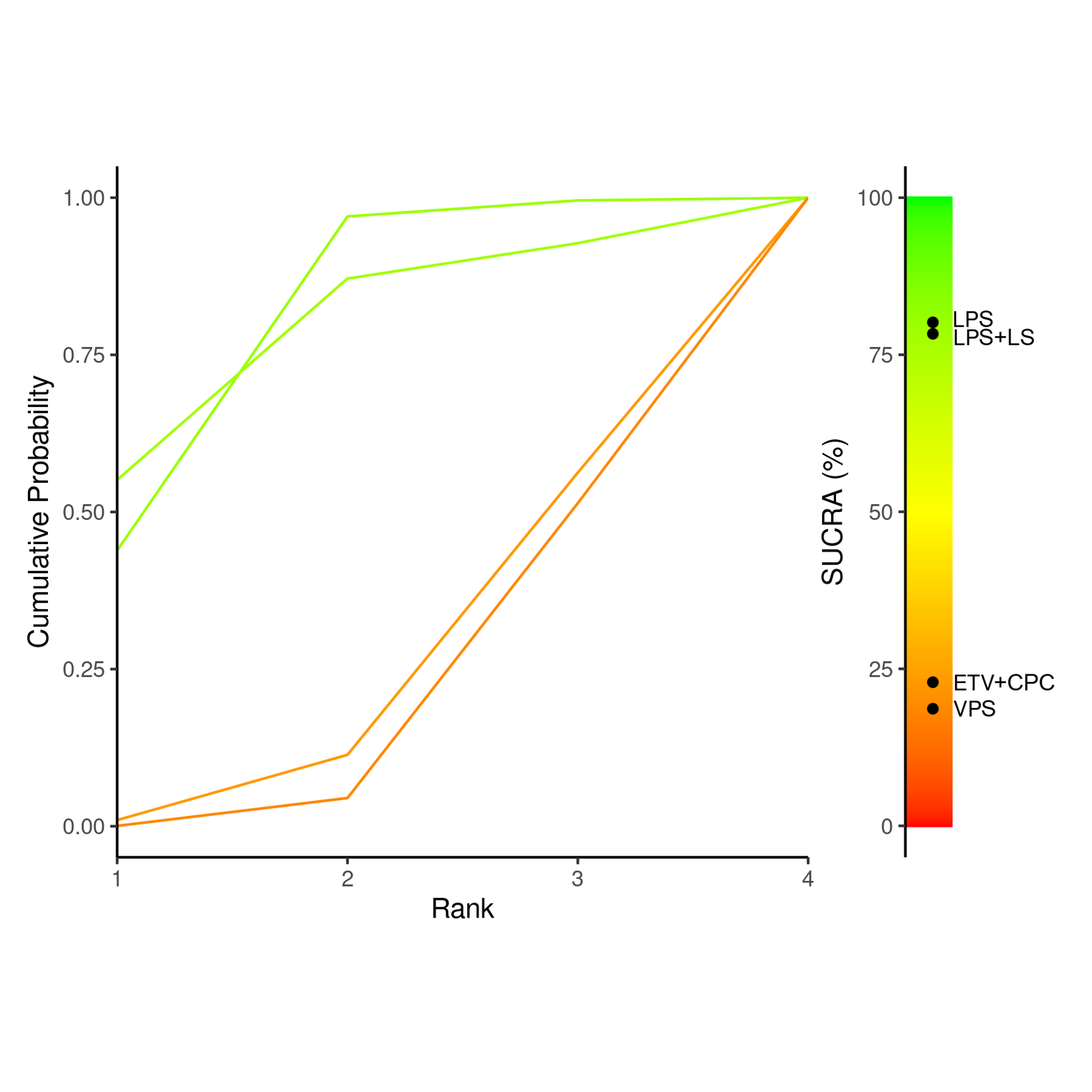


| **Surgical interventions** | **SUCRA score** ^a^ |
| --- | --- |
| **LPS** | **80.15%** |
| LPS+LS | 78.33% |
| ETV+CPC | 28.87% |
| **VPS** | **18.65%** |

^a^ Higher SUCRA values indicated better intervention effects.

**F. Operation time**

**
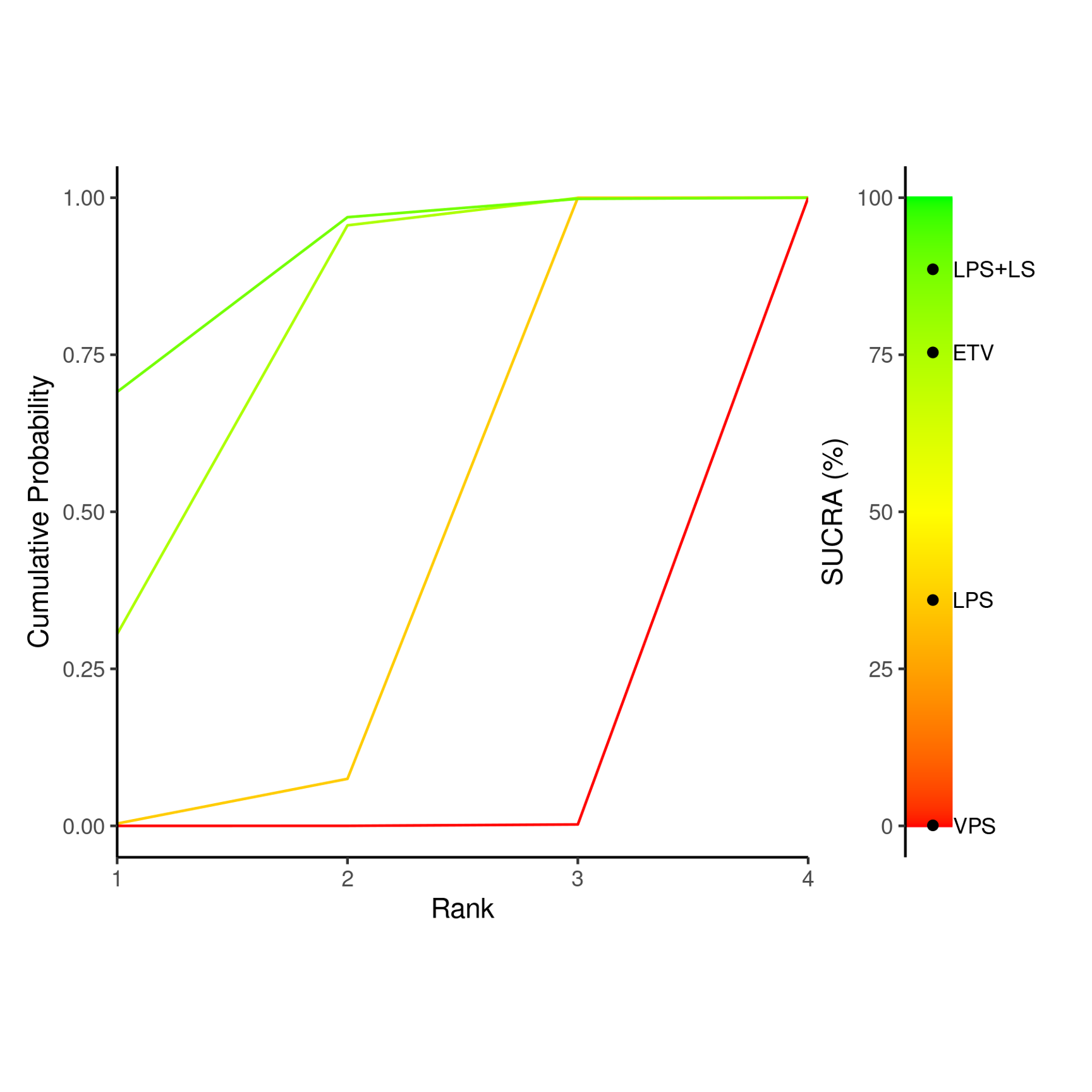
**

| **Surgical interventions** | **SUCRA score** ^a^ |
| --- | --- |
| **LPS+LS** | **88.74%** |
| ETV | 75.27% |
| LPS | 35.89% |
| **VPS** | **0.10%** |

^a^ Higher SUCRA values indicated better intervention effects.

**G. Duration of hospitalization**


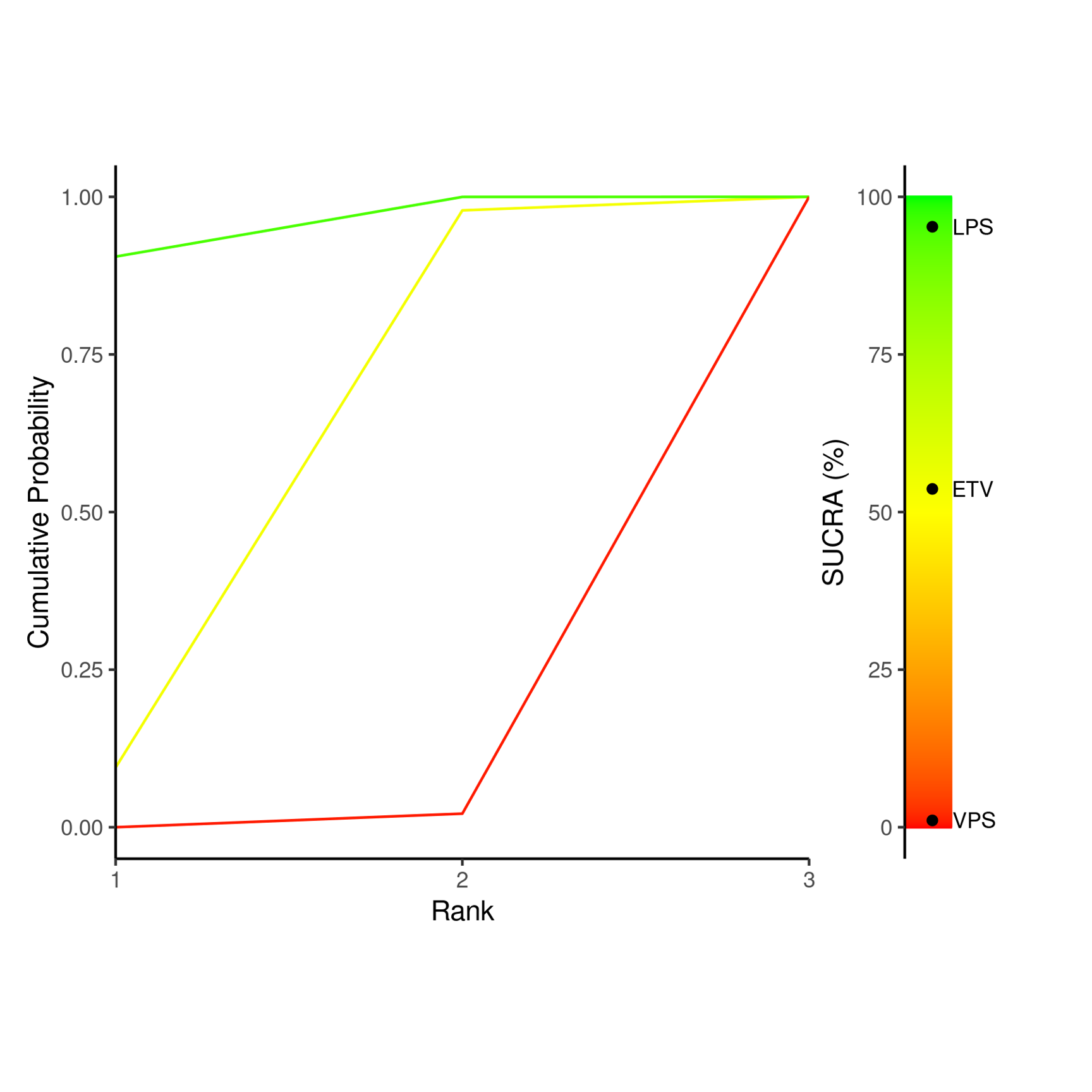


| **Surgical interventions** | **SUCRA score ^a^** |
| --- | --- |
| **LPS** | **95.26%** |
| ETV | 53.67% |
| **VPS** | **1.08%** |

^a^ Higher SUCRA values indicated better intervention effects.

**eFigure 6. Conventional Pairwise Meta-Analyses across Comparisons**

1. **Favorable outcome**

**ETV vs VPS**

**
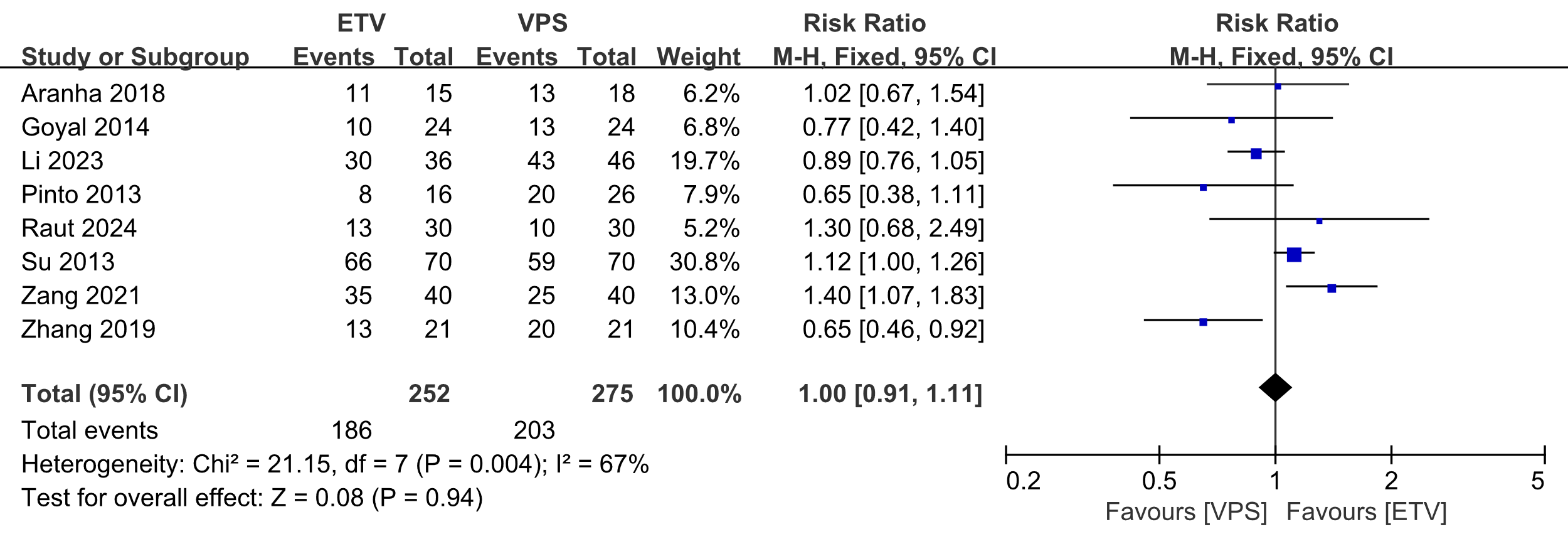
**

**ETV+CPC vs VPS**

**
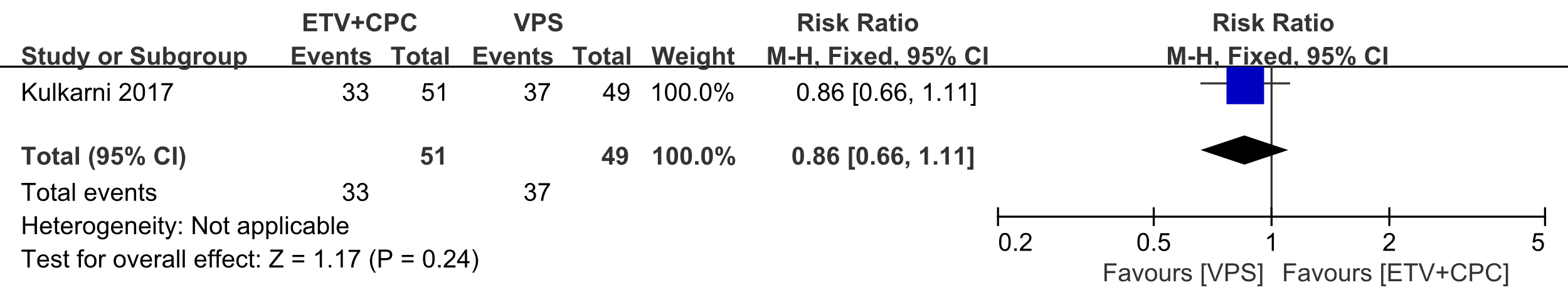
**

**LPS vs VPS**

**
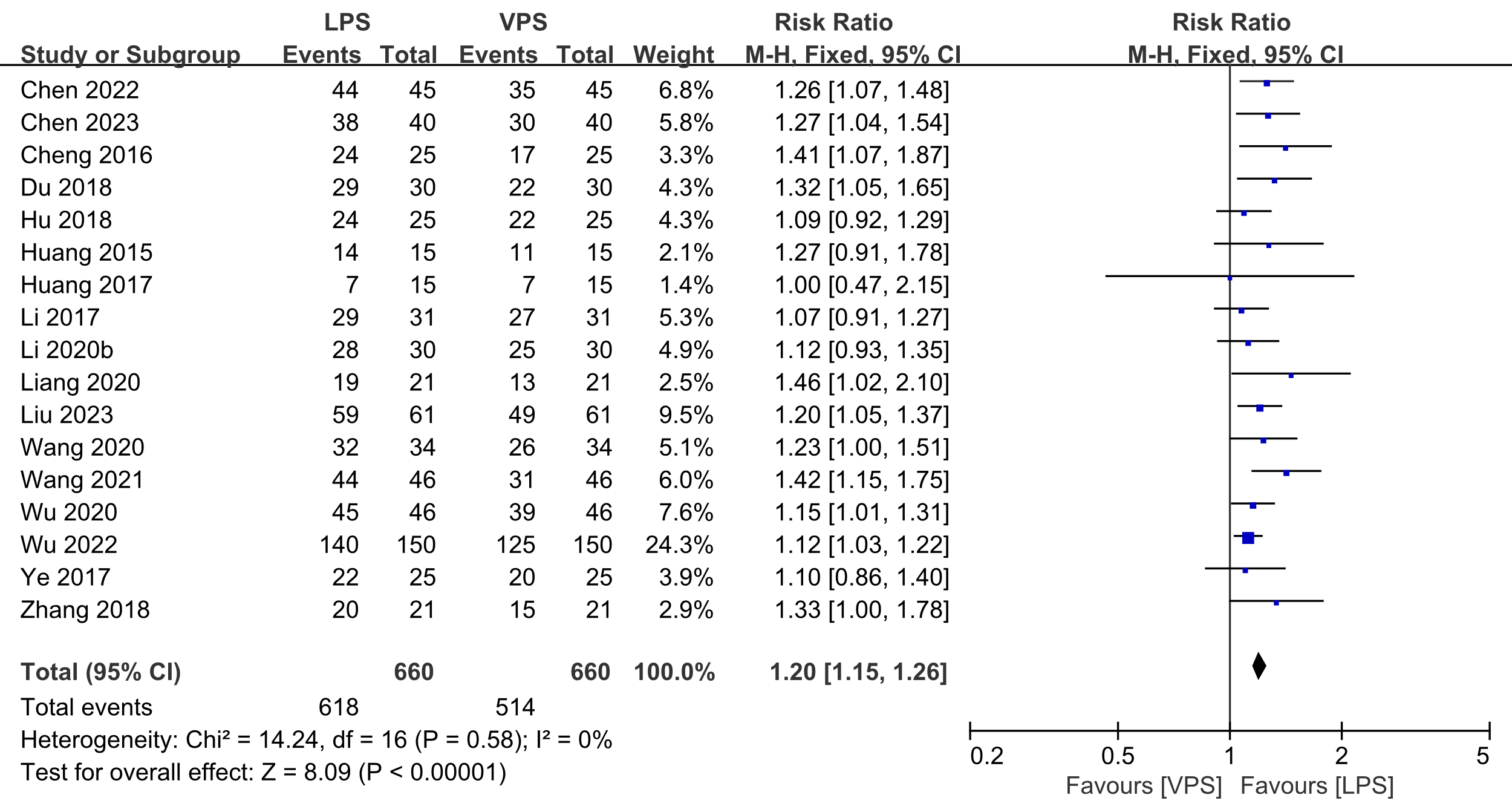
**

**LPS+LS vs VPS**

**
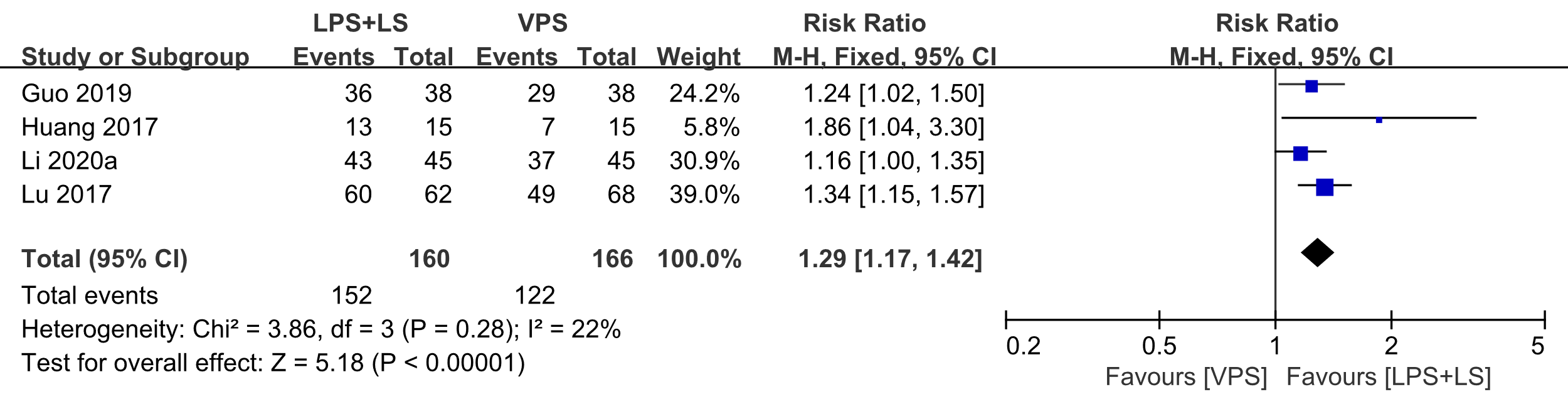
**

**LPS vs LPS+LS**

**
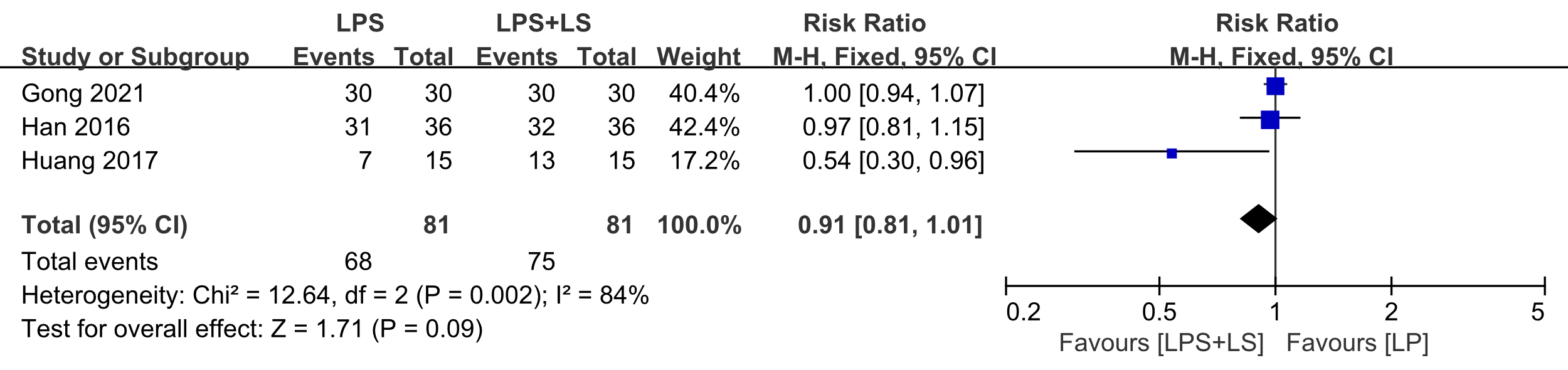
**

**B. Complications**

**ETV vs VPS**

**
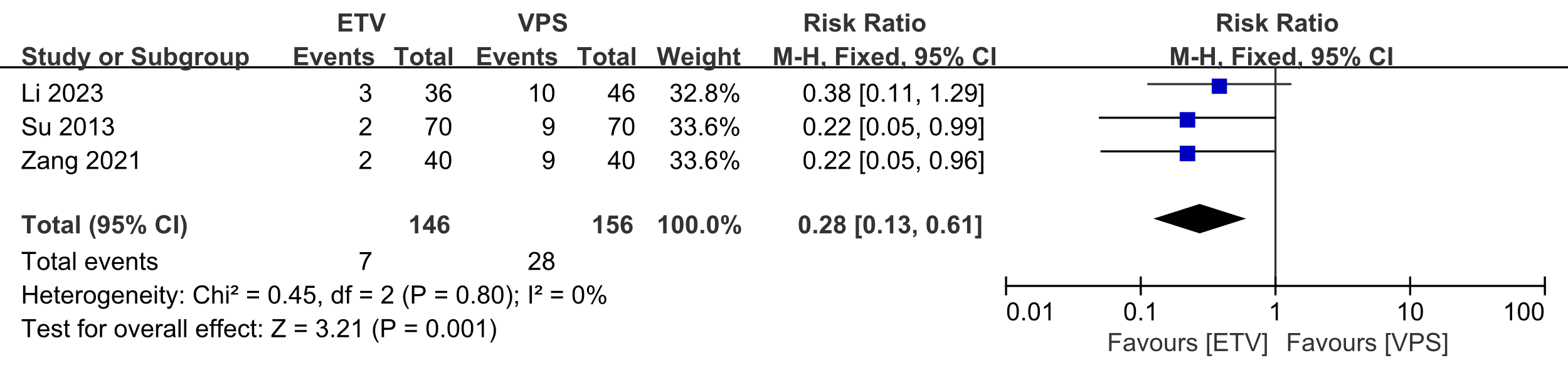
**

**ETV+CPC vs VPS**

**
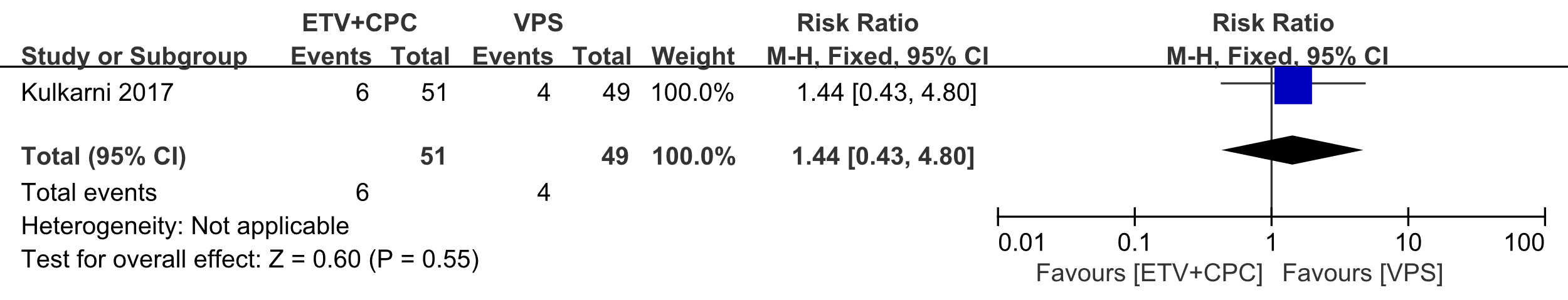
**

**LPS vs VPS**

**
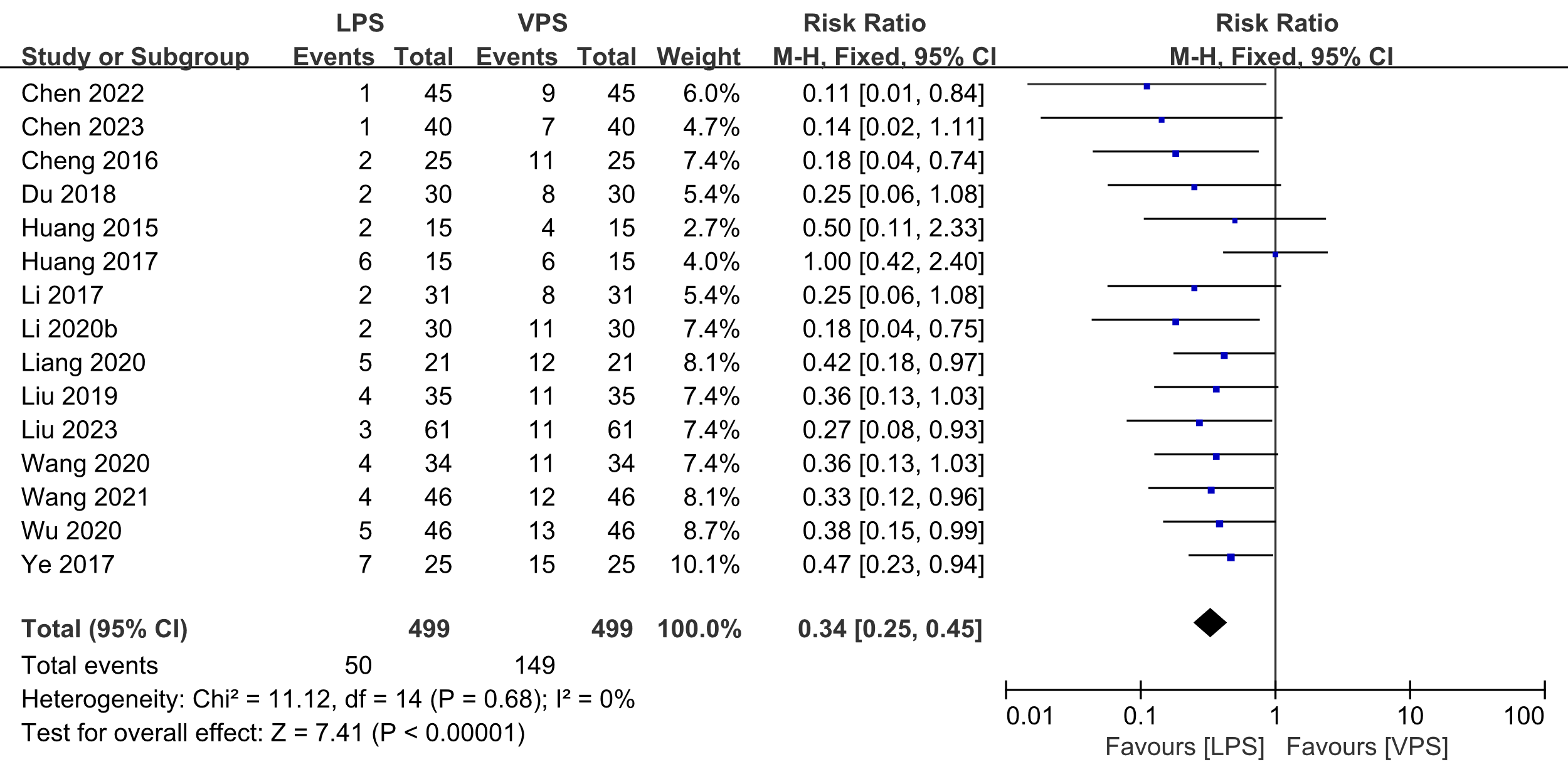
**

**LPS+LS vs VPS
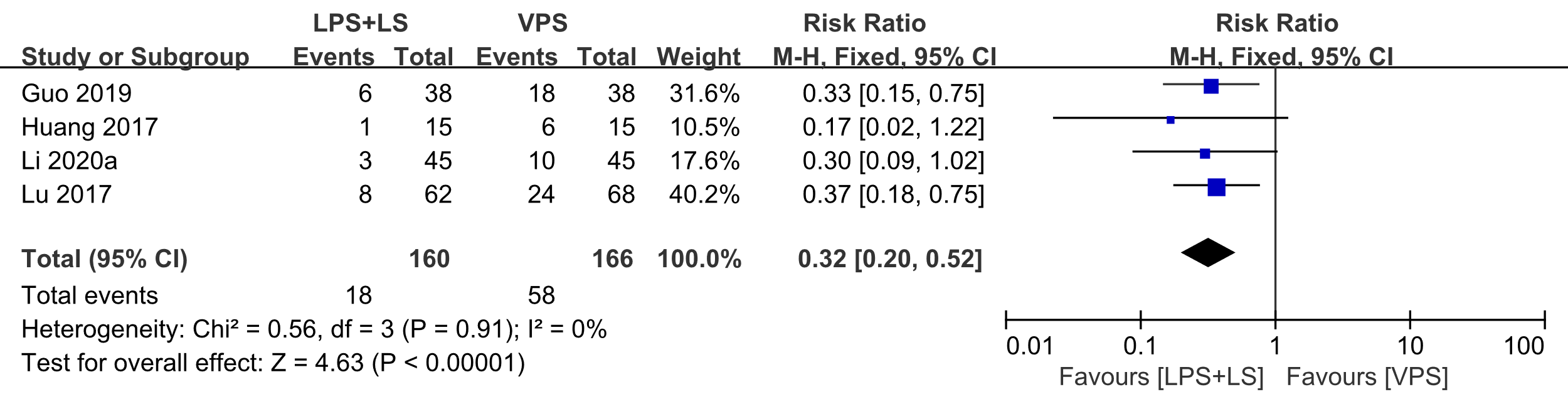
**

**LPS+LS vs LPS**

**
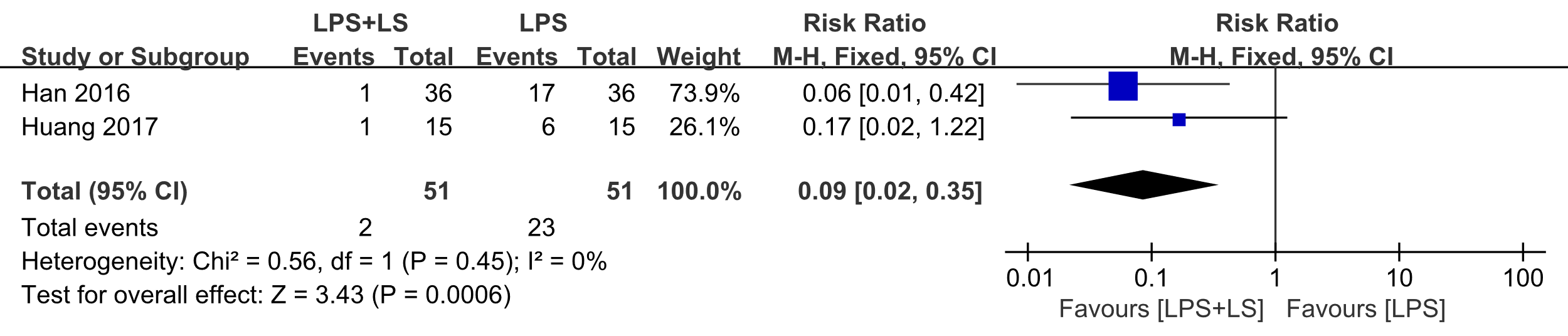
**

**C. Infection**

**ETV vs VPS**

**
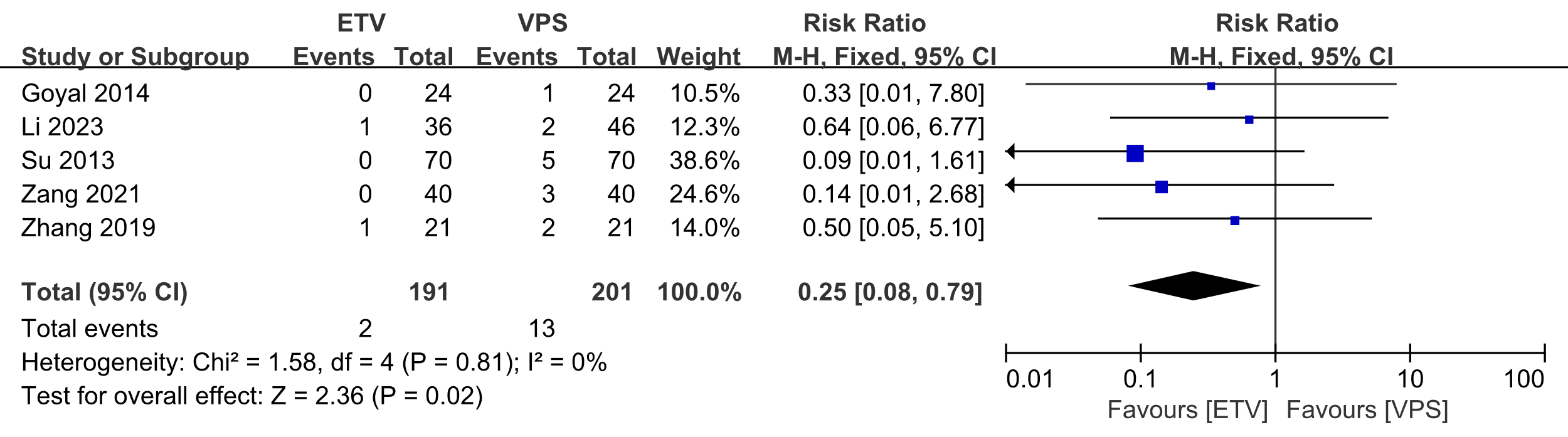
**

**ETV+CPC vs VPS**

**
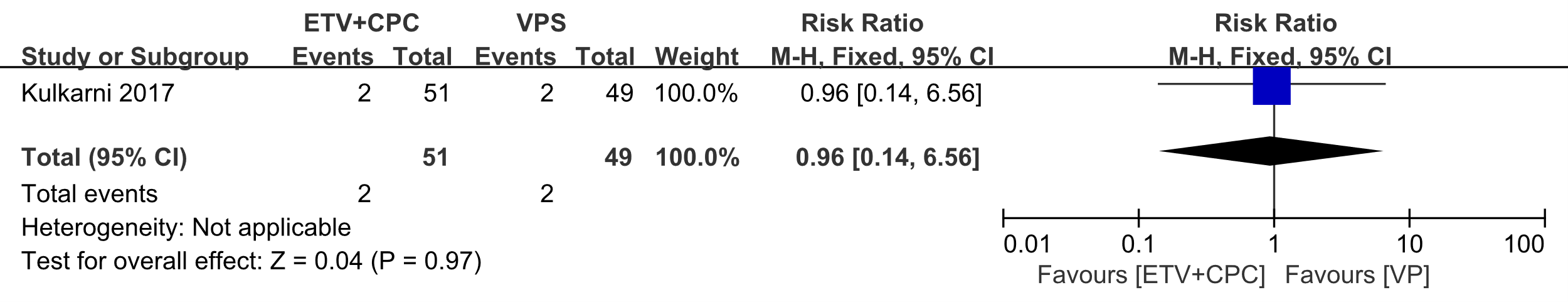
**

**LPS vs VPS**

**
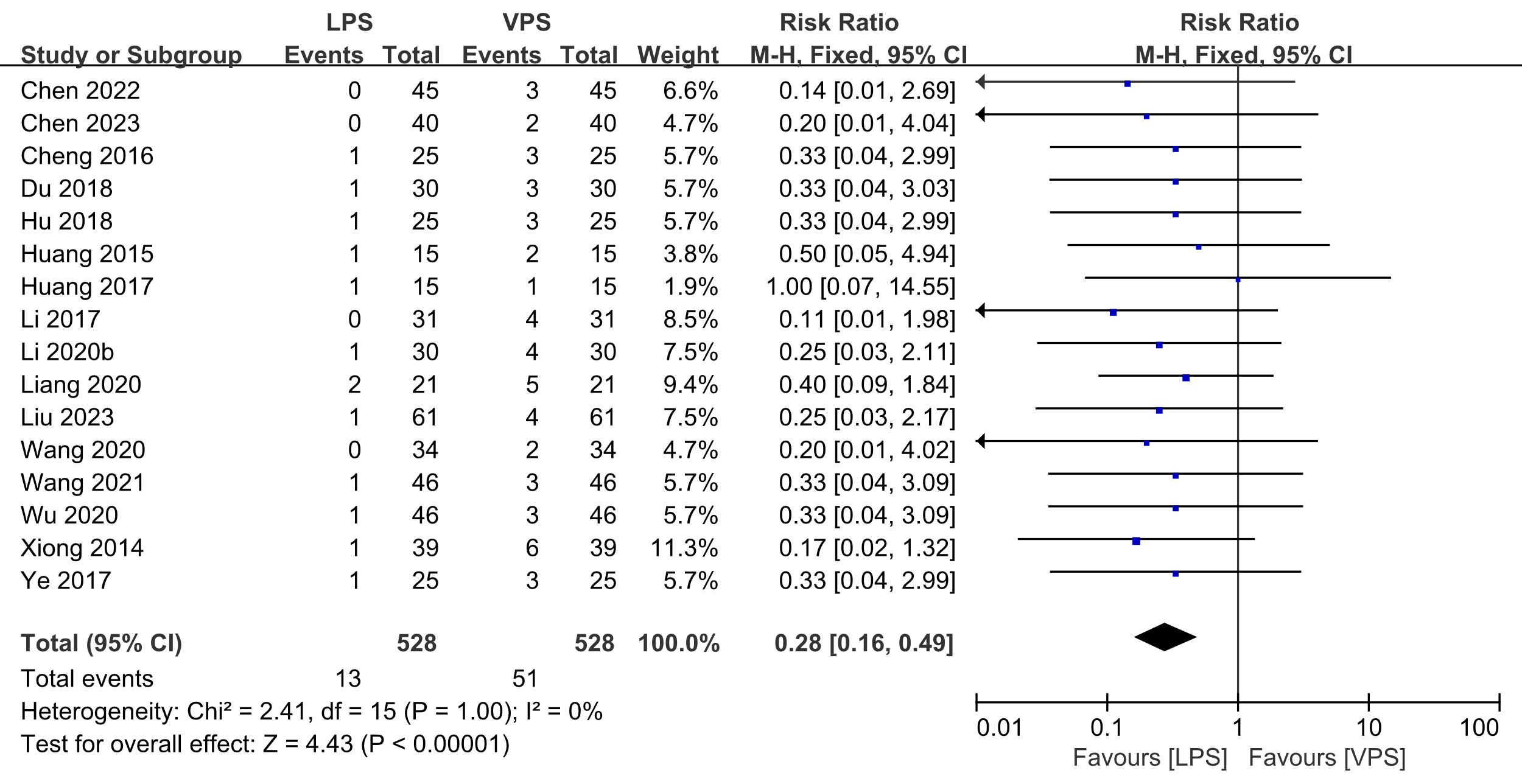
**

**LPS+LS vs VPS**

**
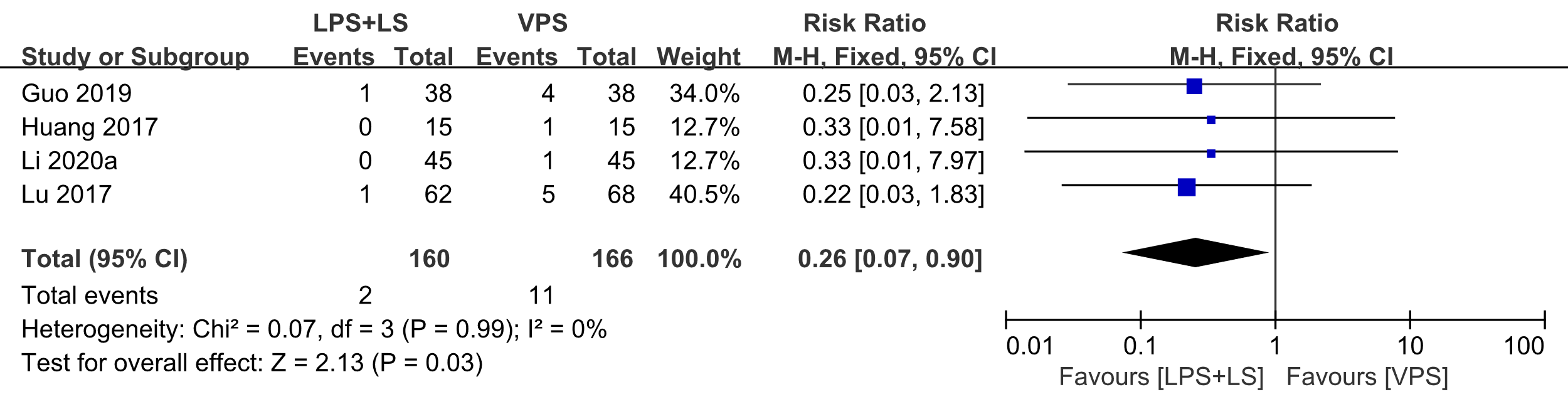
**

**LPS+LS vs LPS**

**
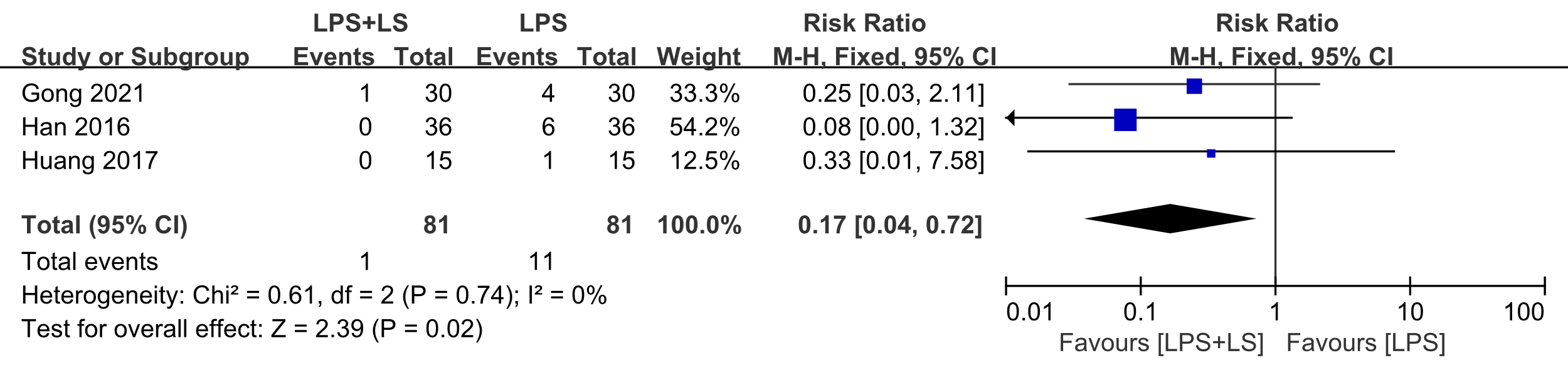
**

**D. Revision**

**ETV vs VPS**

**
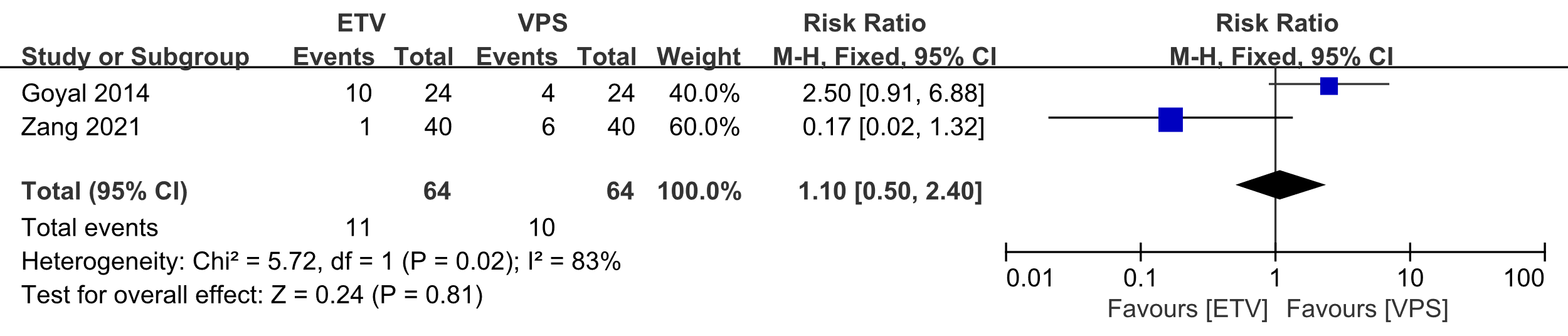
**

**ETV+CPC vs VPS**

**
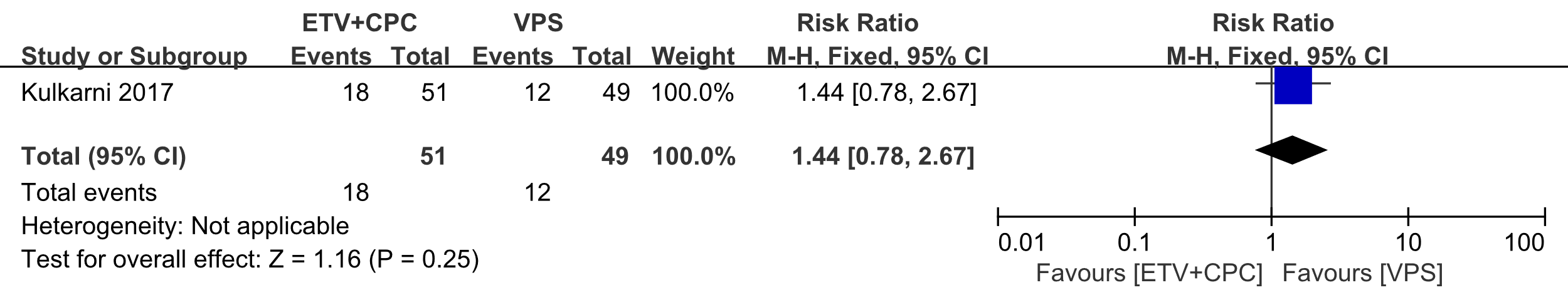
**

**LPS vs VPS**

**
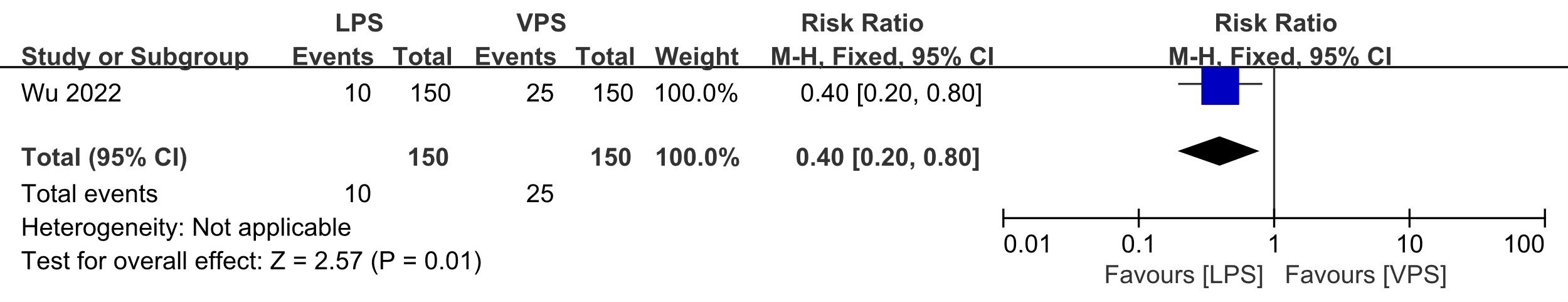
**

**LPS+LS vs VPS**

**
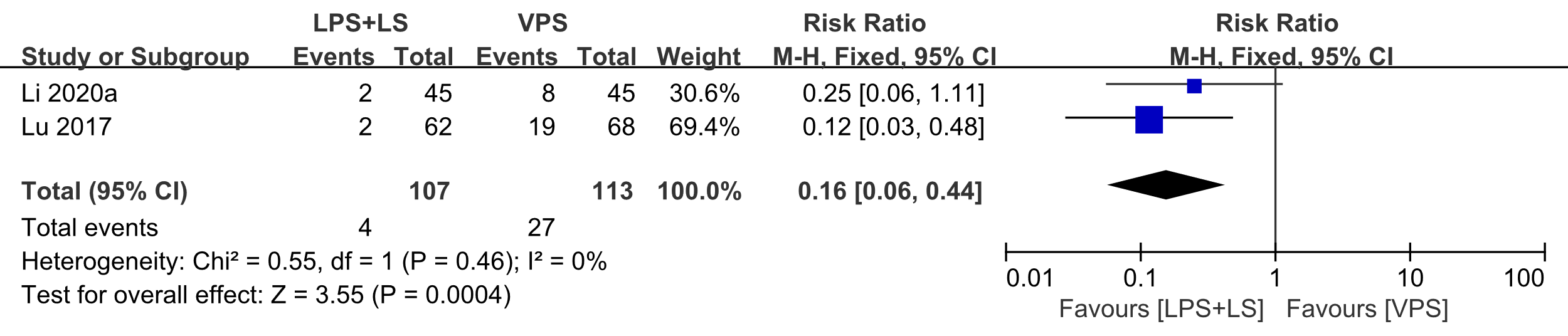
**

**LPS+LS vs LPS**

**
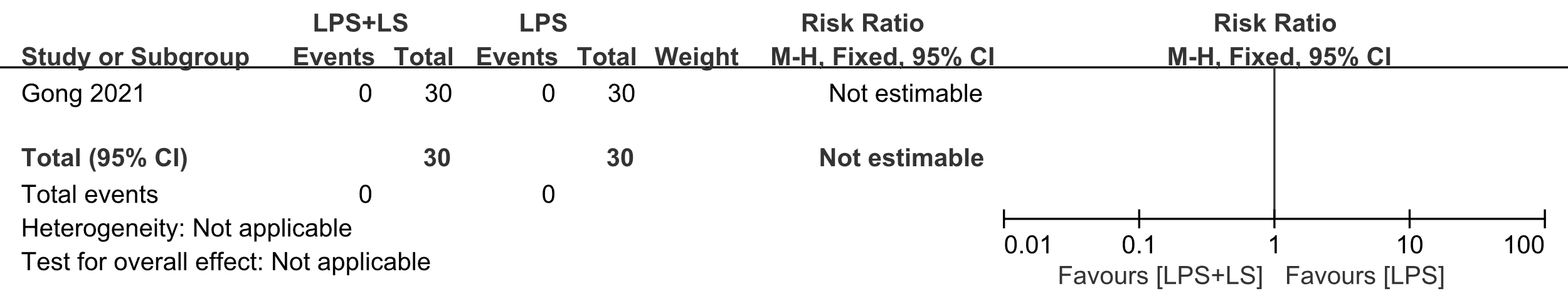
**

**E. Seizures**

**ETV vs VPS**

**
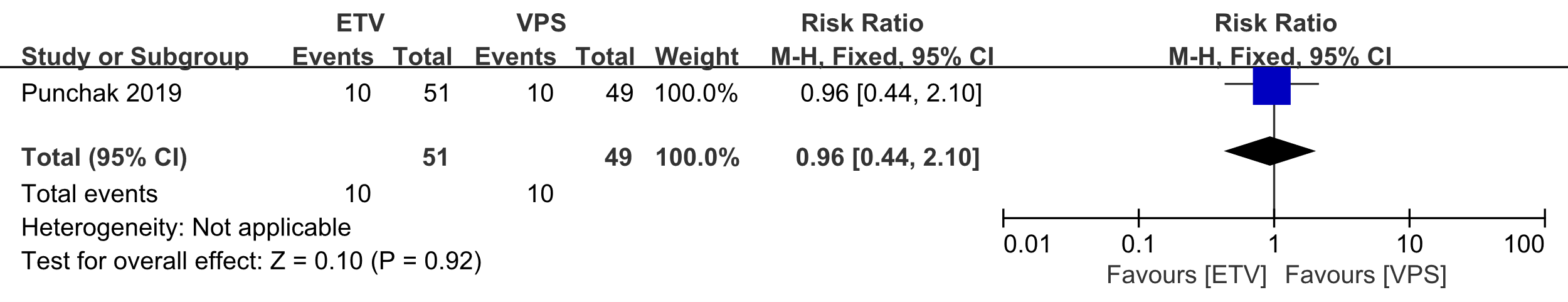
**

**LPS vs VPS**

**
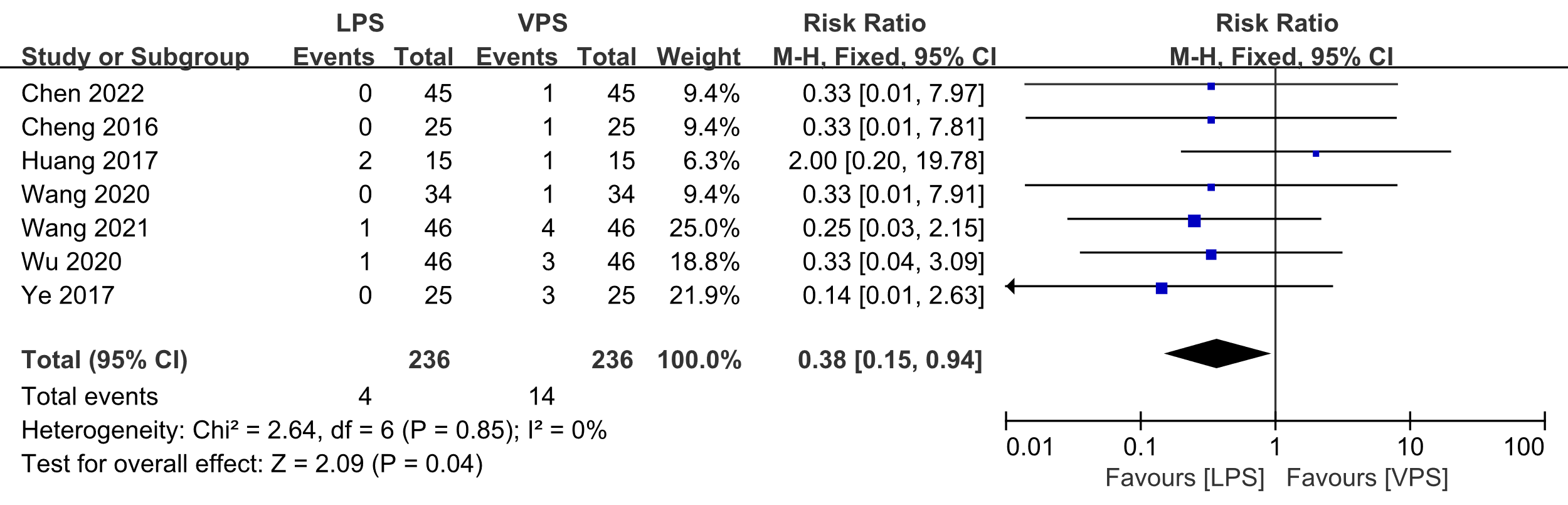
**

**LPS+LS vs VPS**

**
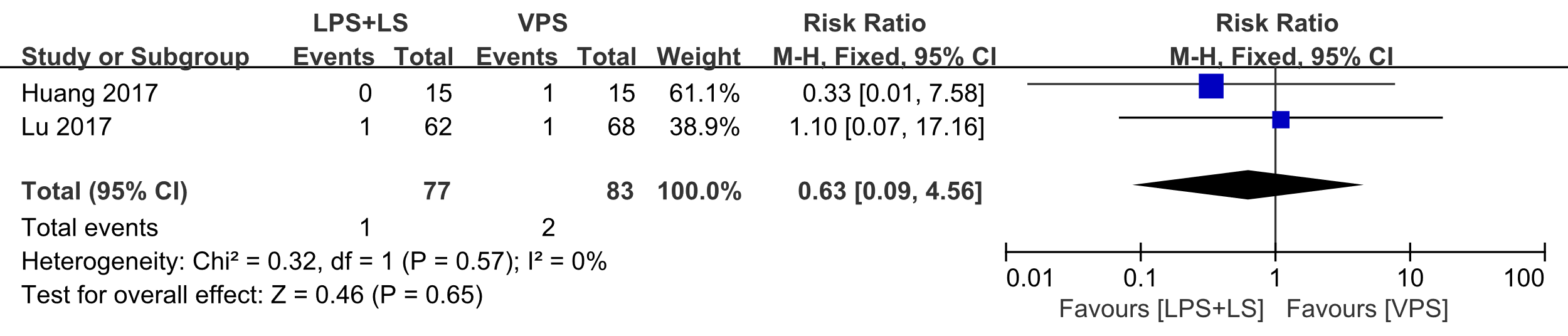
**

**LPS+LS vs LPS**

**
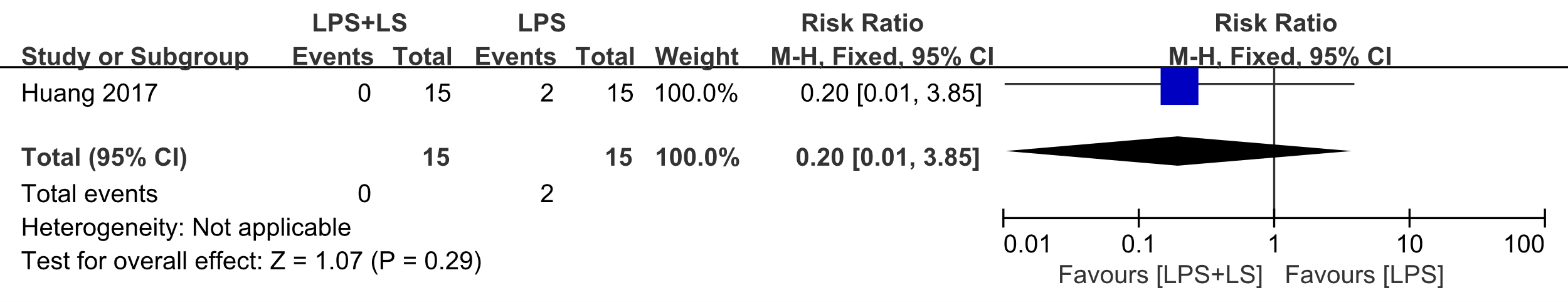
**

**F. Operation time**

**ETV vs VPS**

**
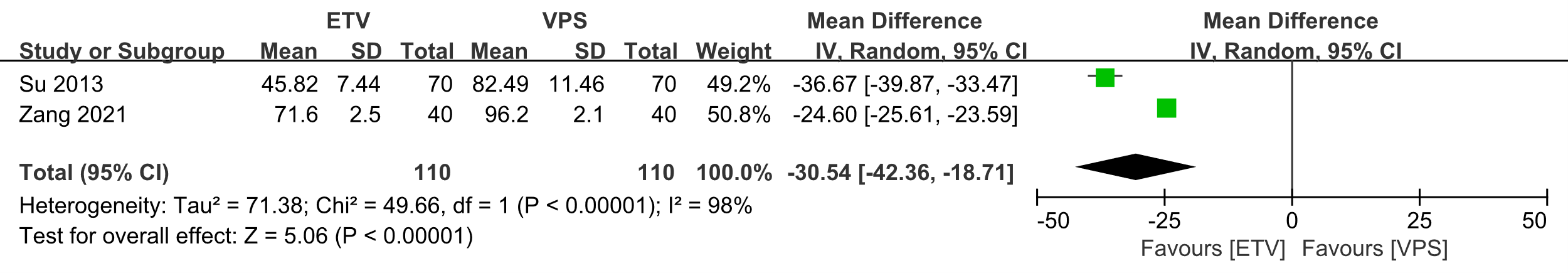
**

**LPS vs VPS**

**
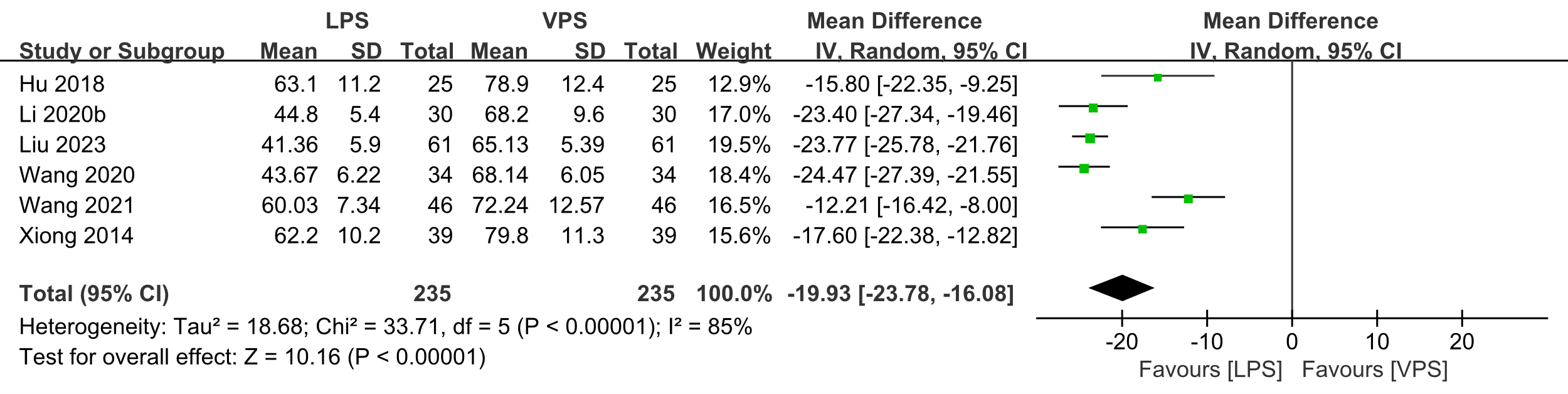
**

**LPS+LS vs LPS**

**
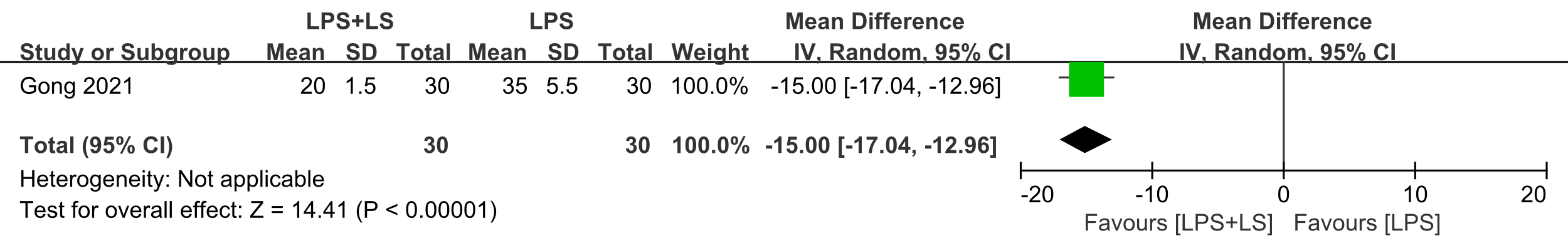
**

**G. Duration of Hospitalisation**

**ETV vs VPS**

**
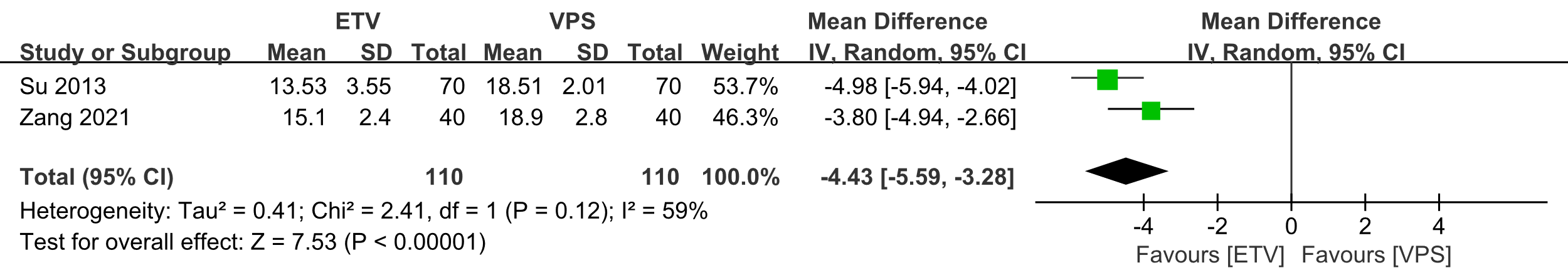
**

**LPS vs VPS**

**
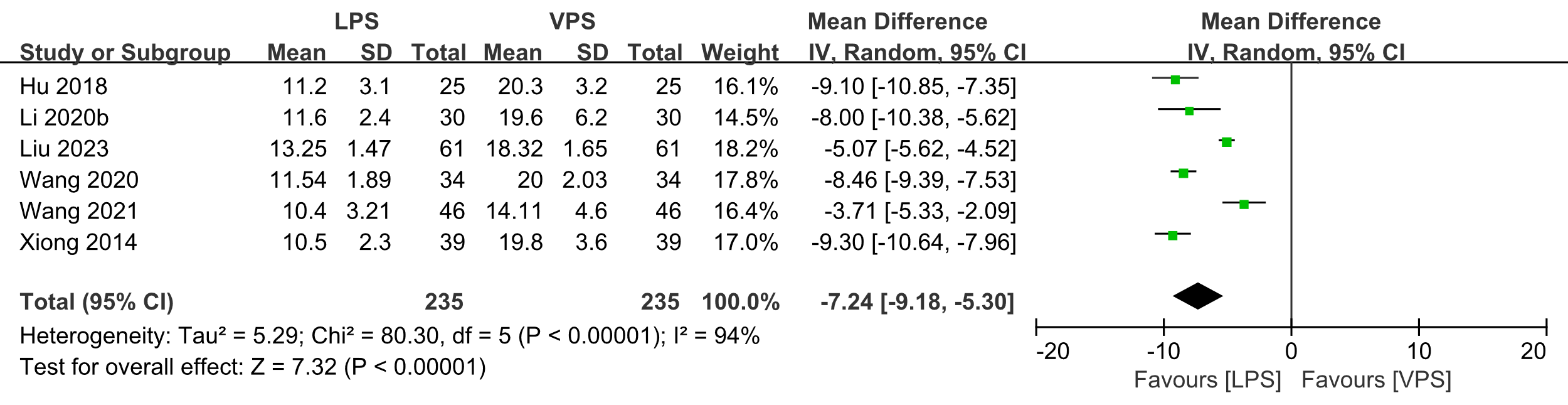
**

**eFigure 7. League Plot for Secondary Outcomes**

**eFigure 8. Sensitivity Analyses for the Network Meta-Analysis of Primary outcomes**

**A. Favorable outcome**

**1. Sensitivity Analyses: random-effects model**

**2. Sensitivity Analyses: adult communicating hydrocephalus**

**3. Sensitivity Analyses: infant communicating hydrocephalus**

**4. Sensitivity Analyses: iNPH**

**5. Sensitivity Analyses: post-infectious hydrocephalus**

**6. Sensitivity Analyses: post-hemorrhagic hydrocephalus**

**7. Sensitivity Analyses: long term follow-up duration (≥ 3 months)**

**B. Complications**

**1. Sensitivity Analyses: random-effects model**

**2. Sensitivity Analyses: adult communicating hydrocephalus**

**3. Sensitivity Analyses: infant communicating hydrocephalus**

**4. Sensitivity Analyses: excluding trials with high risk of bias**

**5. Sensitivity Analyses: post-infectious hydrocephalus**

**6. Sensitivity Analyses: post-****hemorrhagic hydrocephalus**

**7. Sensitivity Analyses: long term follow-up duration (≥ 3 months)**
